# Soil pH is a Key Determinant of Soil Fungal Community Composition in the Ny-Ålesund Region, Svalbard (High Arctic)

**DOI:** 10.3389/fmicb.2016.00227

**Published:** 2016-02-26

**Authors:** Tao Zhang, Neng-Fei Wang, Hong-Yu Liu, Yu-Qin Zhang, Li-Yan Yu

**Affiliations:** ^1^China Pharmaceutical Culture Collection, Institute of Medicinal Biotechnology, Chinese Academy of Medical Sciences and Peking Union Medical CollegeBeijing, China; ^2^Key Lab of Marine Bioactive Substances, First Institute of Oceanography, State Oceanic AdministrationQingdao, China

**Keywords:** soil fungi, fungal community composition, arctic tundra, soil properties, high-throughput sequencing

## Abstract

This study assessed the fungal community composition and its relationships with properties of surface soils in the Ny-Ålesund Region (Svalbard, High Arctic). A total of thirteen soil samples were collected and soil fungal community was analyzed by 454 pyrosequencing with fungi-specific primers targeting the rDNA internal transcribed spacer (ITS) region. The following eight soil properties were analyzed: pH, organic carbon (C), organic nitrogen (N), ammonium nitrogen (NH_4_^+^-N), silicate silicon (SiO_4_^2-^-Si), nitrite nitrogen (NO_2_^-^-N), phosphate phosphorus (PO_4_^3-^-P), and nitrate nitrogen (NO_3_^-^-N). A total of 57,952 reads belonging to 541 operational taxonomic units (OTUs) were found. of these OTUs, 343 belonged to Ascomycota, 100 to Basidiomycota, 31 to Chytridiomycota, 22 to Glomeromycota, 11 to Zygomycota, 10 to Rozellomycota, whereas 24 belonged to unknown fungi. The dominant orders were Helotiales, Verrucariales, Agaricales, Lecanorales, Chaetothyriales, Lecideales, and Capnodiales. The common genera (>eight soil samples) were *Tetracladium, Mortierella, Fusarium, Cortinarius*, and *Atla*. Distance-based redundancy analysis (db-rda) and analysis of similarities (ANOSIM) revealed that soil pH (*p* = 0.001) was the most significant factor in determining the soil fungal community composition. Members of Verrucariales were found to predominate in soils of pH 8–9, whereas Sordariales predominated in soils of pH 7–8 and Coniochaetales predominated in soils of pH 6–7. The results suggest the presence and distribution of diverse soil fungal communities in the High Arctic, which can provide reliable data for studying the ecological responses of soil fungal communities to climate changes in the Arctic.

## Introduction

The Arctic tundra covers approximately 5% of Earth’s land surface (Nemergut et al., 2005) and is one of the most extreme environments on Earth, characterized by low temperatures, frequent freeze-thaw and wet-dry cycles, and low organic matter content. The Arctic tundra is particularly sensitive and vulnerable to global climate change that a rapid rate of climate warming has been observed in recent decades. Arctic average temperatures have increased at almost twice the global average rate over the past 100 years (Solomon, 2007). Some marked environmental changes in the Arctic have been observed by researchers, such as retreat of glaciers, melting of permafrost and sea ice, changes of vegetation, alteration of nutrient cycling and energy flow (Overpeck et al., 1997; Schuur et al., 2009; Pearson et al., 2013).

Arctic microbes can be viewed both as sentinels and amplifiers of global change (Vincent, 2010). Understanding the structure of Arctic soil microbial communities is essential for predicting the response of the Arctic tundra to climate change (Blaud et al., 2015a). Along with soil bacteria, fungi play important roles in driving mineral and energy cycles in Arctic soil environments. They can function as mutualists (mycorrhizae, endophytes, lichens) and decomposers and may affect the carbon balance of terrestrial ecosystems subjected to climate change (Timling and Taylor, 2012). Hitherto, a few studies carried out provide only limited insight to fungal diversity in the Arctic soils. To the best of our knowledge, soil fungal diversity has been reported from different Arctic regions, including Franz Joseph Land (High Arctic) (Bergero et al., 1999), Svalbard (High Arctic) (Robinson et al., 2004; Kurek et al., 2007; Geml et al., 2012; Singh et al., 2012; Ali et al., 2013; Blaud et al., 2015b), Toolik Lake site in Alaska (Low Arctic) (Wallenstein et al., 2007; Deslippe et al., 2012), Greenland (Low and High Arctic) (Meyling et al., 2012), Siberian tundra (High Arctic) (Gittel et al., 2014).

Most of these previous studies focused on the diversity of cultured free-living soil fungi in the Arctic using traditional isolation methods (Bergero et al., 1999; Robinson et al., 2004; Kurek et al., 2007; Meyling et al., 2012; Singh et al., 2012; Ali et al., 2013). However, the cultivation-based methods likely do not reflect the actual diversity of soil fungi because of their selectivity. Additionally, a few studies surveyed soil fungi in the Arctic using conventional DNA-based molecular methods (e.g., DGGE, cloning approaches, q-PCR) (Wallenstein et al., 2007; Deslippe et al., 2012; Geml et al., 2012; Blaud et al., 2015b), which are relatively low taxonomic resolution techniques. In recent years, diversity, and community composition of soil fungi and other microbes in the Arctic tundra have been revealed by high-throughput sequencing (Gittel et al., 2014), which can significantly enhance the characterization of fungal diversity compared to traditional methods. Whilst the previous findings have improved our understanding of soil fungal diversity in the Arctic tundra, the environmental factors shaping the soil fungal community composition in the Arctic, especially High Arctic, are not well understood.

Svalbard is an Arctic archipelago, which is geographically isolated from mainland Eurasia and entirely within the High Arctic Zone. About 59% of its land area is covered by glaciers (Liestøl, 1993). Accordingly, the soil environment in Svalbard is a good representation for the study of the soil fungal communities in the High Arctic. Moreover, no pyrosequencing study has yet been carried out to provide insight to the fungal communities that inhabit soil samples from Svalbard (High Arctic). The aim of this study was to use 454 pyrosequencing to investigate the soil fungal communities in the Ny-Ålesund Region (Svalbard, High Arctic) to address the following questions: (1) What are soil fungal diversity and community composition in this High Arctic region? (2) What is the key environmental factor that determines the soil fungal community structure in this region? (3) How does the key factor affect soil fungal taxonomic groups in this region?

## Materials and Methods

### Study Sites and Sample Collection

The study site was located in the Ny-Ålesund Region (78°55′ N, 11°56′ E), which is on the west coast of Spitsbergen, the largest island of the Svalbard archipelago. It has the lowest temperatures during February, at an average of - 14°C, and a high average of 5°C during July ^1^. The tundra around Ny-Ålesund experiences a short growing season (June to August) when the temperature typically rises only a few degrees above the freezing point. The local area has a silty clay and sand gley soil that has a thin organic soil cover. The soil thawing begins in early June, and the thaw depths reach their maximum (1.6–2.0 m) in the beginning of September and remain approximately constant until at least the mid of the month (Zhu et al., 2012).

Sampling was performed during China’s Arctic expedition in July 2013. The sample collection was under ethical approval of the Svalbard Science Forum, Kings Bay As, and the Chinese Arctic and Antarctic Administration (CAA), the State Oceanic Administration (SOA) of China. Surface soil samples from bare ground without vegetation (∼10 cm^2^) were collected from the top 5 cm at each sampling sites using a sterile shovel. A total of thirteen samples were collected and directly put into TWIRL’EM sterile sampling bags (Labplas Inc., Sainte-Julie, QC, Canada). Soil samples were placed in centrifuge tubes at -20°C in the Yellow River Station (China) for about 20 days and taken to the home laboratory in China by plane. Soil samples were then frozen at -80°C until nucleic acid extraction. The locations of the thirteen soil samples are shown in **Table 1**.

**Table 1:** Locations and geochemical properties of 13 soil samples investigated in the present study.

Sampling Code	Coordinates (N/E)	Altitude (m)	pH	Organic C (%)	Organic N (%)	NH_4_^+^-N (μg/g)	NO_2_^-^-N (μg/g)	NO_3_^-^-N (μg/g)	PO_4_^3-^-P (μg/g)	SiO_4_^2-^-Si (μg/g)
S10.3	78°54′41″N/11°58′35″E	39	6.00	5.04	1.12	25.016	0.084	0.449	0.055	2.099
S12.3	78°57′58″N/12°03′30″E	44	6.44	4.23	0.41	1.537	0.143	4.580	0.029	0.725
S12.1	78°57′58″N/12°03′31″E	45	6.45	2.39	0.26	1.146	0.154	1.024	0.025	0.679
S8.3	78°57′36″N/11°36′04″E	35	6.88	11.04	1.41	88.325	0.119	0.696	0.134	2.941
S8.2	78°57′38″N/11°35′57″E	34	6.94	12.15	1.27	12.959	0.148	0.794	0.103	3.073
S17.1	78°55′04″N/11°56′33″E	21	7.71	2.51	0.26	2.131	0.792	4.601	0.033	3.699
S17.2	78°55′04″N/11°56′34″E	21	7.83	0.29	0.30	7.619	0.148	0.770	0.051	4.699
S18.2	78°55′00″N/11°51′37″E	64	7.93	4.84	0.41	33.838	0.048	0.256	0.009	4.174
S17.3	78°55′04″N/11°56′32″E	21	7.96	3.65	0.21	3.413	0.171	0.699	0.041	7.427
S16.1	78°53′53″N/12°04′07″E	97	8.33	0.25	0	11.824	0.112	0.538	0.048	8.277
S16.2	78°53′53″N/12°04′06″E	97	8.45	0.58	0.15	10.425	0.110	0.568	0.030	5.635
S18.1	78°54′52″N/11°51′25″E	77	8.48	0.55	0.13	11.378	0.168	0.952	0.001	2.709
S10.1	78°54′48″N/11°50′44″E	87	8.54	0.42	0.15	7.379	0.245	1.256	0.062	7.904

### Physical and Chemical Properties of Soil

A total of eight soil properties were assessed, including pH, organic carbon (C), organic nitrogen (N), ammonium nitrogen (NH_4_^+^-N), silicate silicon (SiO_4_^2-^-Si), nitrite nitrogen (NO_2_^-^-N), phosphate phosphorus (PO_4_^3-^-P) and nitrate nitrogen (NO_3_^-^-N). Soil pH was measured by adding 10 ml of distilled water to 4 g of soil and recording pH using a pH electrode (PHS-3C, Shanghai REX Instrument Factory, Shanghai, China). Analysis of organic C and organic N was performed using an Elemental Analyzer (EA3000, Euro Vector SpA, Milan, Italy). The other five properties were analyzed using a High Performance Microflow Analyzer (QuAAtro, SEAL Analytical GmbH, Norderstedt, Germany).

### DNA Extraction and PCR Amplification

Genomic DNA was extracted from an aliquot of 0.25 g of wet soil from each sample using a PowerSoil DNA Isolation Kit (MO BIO Laboratories, San Diego, CA, USA) according to the manufacturer’s instructions (three replicates for each sample). The combined DNA extracts were then used for the subsequent PCR and sequencing experiments. The primer pair ITS1F (5′-CTTGGTCATTTAGAGGAAGTAA-3′) and ITS4 (5′-TCCTCCGCTTATTGATATGC-3′) (White et al., 1990) was used for amplifying the rDNA internal transcribed spacer (ITS, ITS1-5.8S-ITS2) region. The PCR amplification was performed using the Amplicon Fusion Primers 5′-A-x-ITS1F-3′ and 5′-B-ITS4-3′, where A and B represent the pyrosequencing adaptors (CCATCTCATCCCTGCGTGTCTCCGACGACT and CCTATCCCCTGTGTGCCTTGGCAGTCGACT, respectively) and x represents an 8 bp-tag for sample identification. The 20 μl reaction mixture contained 10 ng of template DNA, 4 μl of 5 × buffer (50 M Tris-HCl, pH 8.3–8.8), 2 μl of 2.5 nM dNTPs, 0.8 μl of Fastpfu Polymerase, 2 μM of each primer and ultra-pure sterilized water. The PCR amplification consisted of an initial denaturation at 95°C for 2 min, followed by 30 cycles of denaturation at 95°C for 30 s, annealing at 55°C for 30 s, and extension at 72°C for 30 s, and a final extension at 72°C for 5 min.

### 454 Pyrosequencing

After purification using the AxyPrep DNA Gel Extraction Kit (Axygen Biosciences, Inc., USA) and quantification using QuantiFluor-ST (Promega Corporation, USA), equimolar mixtures of multiple amplicons were used for pyrosequencing on a Roche 454 GS FLX+ Titanium platform (Roche 454 Life Sciences, US) according to standard protocols. The raw sequences were deposited in the NCBI sequencing read archive (SRA) under Accession No. SRP067367.

### Pyrosequencing Data Processing

Raw sequence data from pyrosequencing were processed using QIIME 1.8.0 software (Caporaso et al., 2010). Briefly, the sequence libraries were split and denoised to avoid diversity overestimation caused by sequencing errors, such as sequences with an average quality score <20 over a 50 bp sliding window, sequences shorter than 200 bp, sequences with homopolymers longer than six nucleotides, and sequences containing ambiguous base calls or incorrect primer sequences. Operational Taxonomic Units (OTUs) were clustered with a 97% similarity cutoff using UPARSE (Edgar, 2013), and chimeric sequences were identified and removed using UCHIME (Edgar et al., 2011). Singleton OTUs, and OTUs that were assigned to non-fungal organisms and had unreliable BLAST matches (max score below 200 or aligned query sequence below 200 bp) were removed. These OTUs were then used as a foundation for calculating alpha-diversity and beta-diversity metrics using QIIME 1.8.0 software (Caporaso et al., 2010).

### Statistical Analyses

Sequences representing the OTUs were subjected to BLASTn search in UNITE^2^ to determine their taxonomic affiliation and GenBank^3^ to determine their originally reported habitat. Statistical analyses of the OTU diversity of each soil sample via Chao1, Good’s coverage estimator, and Shannon’s index (*H*′) were performed using QIIME 1.8.0 software (Caporaso et al., 2010). The abundance-based Bray–Curtis similarity coefficient was used to examine the dissimilarity of different soil samples. The relevance of environmental factors in explaining the distribution patterns of fungal communities in different soil samples was analyzed by distance-based redundancy analysis (db-RDA) using the R 3.1.1 statistical software. An analysis of similarities (ANOSIM) was performed using QIIME 1.8.0 software (Caporaso et al., 2010) to determine whether different soil types had significantly different fungal communities. A linear discriminant analysis effect size (LEfSe) method was used to identify the significantly different fungal groups in different soil types (Segata et al., 2011).

## Results

### Physical and Chemical Properties of Soil

The pH of soil samples collected were greatly different form each other, which were in the range of 6.00 to 8.54. The organic carbon contents ranged from 0.25 to 12.15%, whereas organic nitrogen contents were below detection to 1.41%. The ammonium nitrogen (NH_4_^+^-N) concentrations ranged from 1.146 to 88.325 μg/g soil, which was much higher than the concentrations of nitrate nitrogen (NO_3_^-^-N, 0.256–4.601 μg/g soil) and nitrite nitrogen (NO_2_^-^-N, 0.048–0.792 μg/g soil) at all sampling sites. The concentrations of phosphate phosphorus (PO_4_^3-^-P) were negligible (0.001–0.134 μg/g soil). The concentrations of silicate silicon (SiO_4_^2-^-Si) were from 0.679 to 8.277 μg/g soil (**Table 1**). In addition, significant correlations were found between pH and some soil properties, including silicate silicon (*r* = 0.768, *p* < 0.01), organic C (*r* = -0.596, *p* < 0.05), and organic N (*r* = -0.666, *p* < 0.05) (Supplementary Table S1).

### Soil Fungal Diversity and Community Structure

The raw data from the 13 soil samples consisted of 106,003 sequence reads, of which 57,952 reads representing 541 fungal OTUs were included in the final matrix after quality filtering and processing. The number of OTUs in different soil samples ranged from 56 to 214 (**Table 2**). The taxonomy of all detected OTUs is listed in Supplementary Table S2. The Chao 1, Good’s coverage estimator and Shannon’s index were used to evaluate and compare the diversity of the fungal communities among different soil samples (**Table 2**). The Good’s coverage estimator ranged from 98.46 to 99.73%, indicating that 454 pyrosequencing captured the dominant phylotypes. The Shannon’s index (*H*′ = 2.73–5.64) indicated that diversity varies among the 13 soil samples.

**Table 2:** Summary data for pyrosequencing data from the 13 soil samples.

Sample code	Valid reads	Trimmed reads	Number of OTUs^#^	Chao 1	Good’s coverage estimator (%)	Shannon’s index
S 10.3	9404	2618	74	86	99.35	3.45
S 12.3	10094	5932	56	86	99.73	2.98
S 12.1	5994	2753	79	111	99.05	2.99
S 8.3	8604	4906	142	153	99.59	4.36
S 8.2	9643	3447	116	141	99.33	4.97
S 17.1	7884	2012	95	128	98.46	4.01
S 17.2	9461	8437	214	252	99.63	5.64
S 18.2	6109	4854	171	204	99.29	5.47
S 17.3	8170	4412	59	89	99.63	2.73
S 16.1	7424	4954	92	109	99.65	4.57
S 16.2	7925	4832	81	88	99.73	4.19
S 18.1	7122	4173	151	189	99.23	5.23
S 10.1	8169	4622	86	105	99.56	4.35

*^#^Defined at the cutoff 3% difference in sequence.*

**FIGURE 1:**
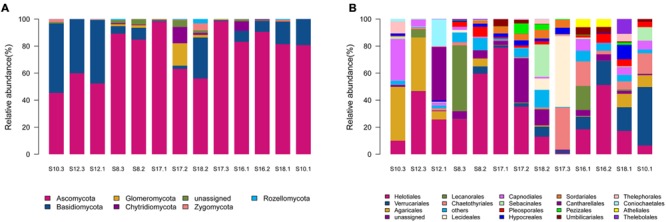
**The relative abundances of different phyla **(A)** and orders **(B)** in the thirteen soil samples in the present study.** The 13 soil samples were arranged in the order of the pH values (from the lowest pH in the left to the highest pH in the right).

Of the 541 OTUs, 343 belonged to Ascomycota, 100 to Basidiomycota, 31 to Chytridiomycota, 22 to Glomeromycota, 11 to Zygomycota, 10 to Rozellomycota, whereas 24 belonged to unknown fungi (Supplementary Table S2). Ascomycota dominated in the 12 soil samples, whereas Basidiomycota dominated in only one sample S10.3 (**Figure 1A**).

Sequences from Ascomycota matched 28 known orders, with Helotiales being the most abundant (16690 reads) and diverse (108 OTUs), followed by Verrucariales (4954 reads, 28 OTUs), Chaetothyriales (3697 reads, 25 OTUs), Capnodiales (2385 reads, 24 OTUs), and Pleosporales (1572 reads, 29 OTUs). Sequences from Basidiomycota matched 15 known orders, with Agaricales (4692 reads, 33 OTUs) and Sebacinales (1842 reads, 14 OTUs) being abundant and diverse. Chytridiomycota sequences were represented by Chytridiales (171 reads, 5 OTUs), Rhizophydiales (272 reads, 5 OTUs) and Olpidiales (355 reads, 2 OTUs). Glomeromycota sequences were represented by Glomerales (18 reads, 1 OTU), whereas Zygomycota sequences were represented by Mortierellales (411 reads, 11 OTUs). As shown in **Figure 1B**, the relative abundance of the major fungal orders varied in the 13 soil samples.

Among the 95 known genera detected in the present study, the most commonly identified genera included *Tetracladium* (3677 reads in 12 samples), *Mortierella* (361 reads in 11 samples), *Fusarium* (242 reads in 11 samples), *Cortinarius* (1226 reads in 10 samples), *Atla* (1496 reads in 9 samples), *Sebacina* (1228 reads in 8 samples), *Nectria* (390 reads in 8 samples), *Cryptococcus* (201 reads in 8 samples), *Inocybe* (138 reads in 8 samples), *Preussia* (152 reads in 8 samples), *Aspergillus* (63 reads in 8 samples), *Thelebolus* (513 reads in 8 samples), *Rhinocladiella* (1909 reads in 8 samples), *Venturia* (226 reads in 8 samples), *Fontanaspora* (29 reads in 8 samples), and *Phoma* (171 reads in 8 samples) (Supplementary Table S2).

In terms of original reported habitats, most of matching sequences with high similarity (≥97%) in GenBank were derived from fungi found in soils the Arctic tundra, including Svalbard and North American Arctic. Additionally, some matching sequences were also reported from other habitats (e.g., plant tissues, glacier ice, sediments) in and beyond the Arctic regions (Supplementary Table S2).

### The Correlation Between Soil Fungal Communities and Geochemical Properties

**FIGURE 2:**
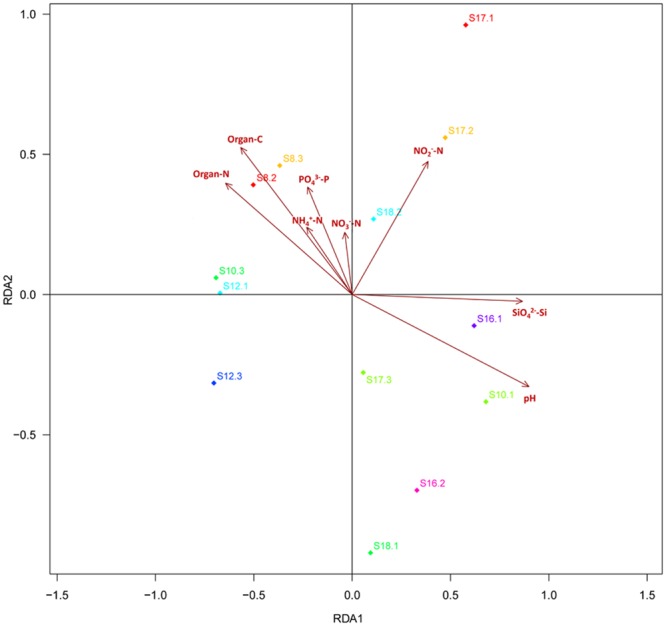
**The db-RDA diagram showing the relationship between the soil properties and fungal community composition.** The thirteen soil samples are labeled with a unique sampling code. Rectangles represent different soil properties.

Distance-based redundancy analysis (db-RDA) (**Figure 2**) and Monte Carlo permutation test (**Table 3**) were performed to examine the relationship between the eight soil geochemical factors and soil community composition. The combination of the eight geochemical factors showed a significant correlation with soil fungal community structure (*F* = 1.175068, *p* = 0.031). These factors explained 70.15% of the soil fungal community variation, while 29.85% of the variation was not explained by any of the selected eight geochemical factors. Among the selected geochemical factors, pH (*r*^2^ = 0.8851, *p* = 0.001) was the most significant geochemical factor that drive the soil fungal communities in this region. The factors SiO_4_^2-^-Si (*r*^2^ = 0.7159, *p* < 0.01), organic C (*r*^2^ = 0.5844, *p* < 0.05), and organic N (*r*^2^ = 0.5576, *p* < 0.05) were also significantly correlated with the soil fungal communities in this region. The other four factors, including NH_4_^+^-N, NO_2_^-^-N, NO_3_^-^-N, and PO_4_^3-^-P were not significantly correlated with soil fungal community composition (**Table 3**).

**Table 3:** A Monte Carlo permutation test of environmental factors and soil fungal community composition.

	RDA1	RDA2	*r*^2^	*P* value
pH	0.931696	-0.363238	0.8851	0.001^∗∗∗^
Organic N	-0.841189	0.540742	0.5576	0.025^∗^
Organic C	-0.725395	0.688333	0.5844	0.014^∗^
NH_4_^-^-N	-0.685736	0.727850	0.1089	0.635
NO_2_^-^-N	0.618917	0.785456	0.3550	0.052
NO_3_^-^-N	-0.180275	0.983616	0.0497	0.789
SiO_4_^2-^-Si	-0.507610	0.861587	0.1951	0.338
PO_4_^3-^-P	0.998889	-0.047119	0.7159	0.004^∗∗^

*^∗^Correlation is significant at the 0.05 level.*

*^∗∗^Correlation is significant at the 0.01 level.*

*^∗∗∗^Correlation is significant at the 0.001 level.*

*p values based on 999 permutations.*

**FIGURE 3:**
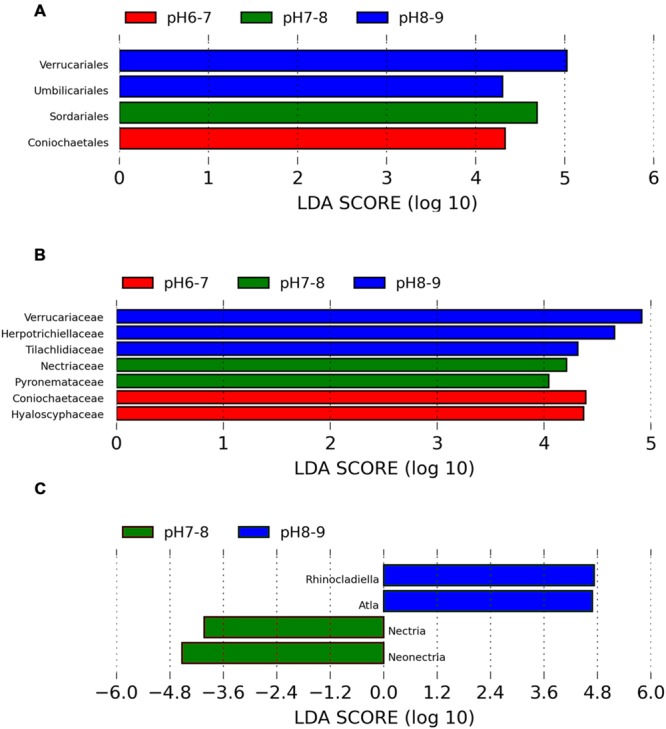
**The Lefse analysis showing the fungal orders **(A)**, families, **(B)**, and genera **(C)** which are significantly different among three soil pH levels**.

**FIGURE 4:**
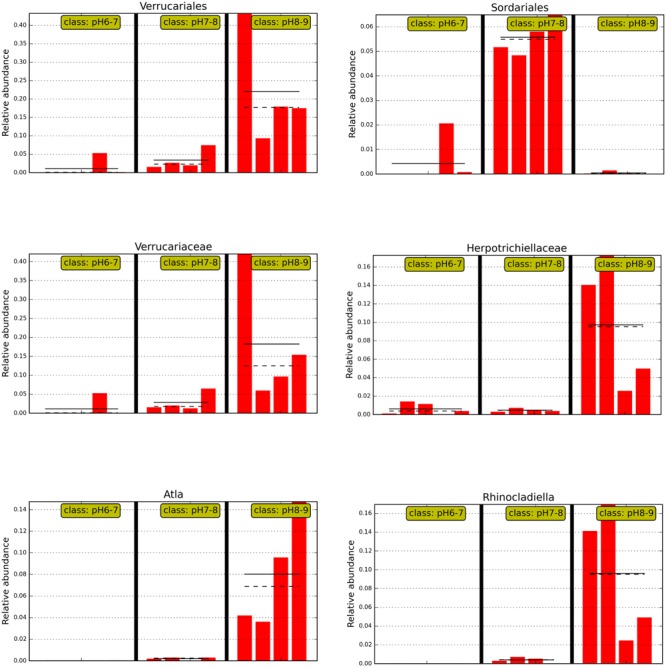
**The abundance histogram of major taxonomic units (relative abundance >2%) which are significantly different among three soil pH levels using Lefse analysis**.

An ANOSIM test (*A* = 0.3474, *p* = 0.011) supported that the three soil types with different pH levels (i.e., pH 8–9, pH 7–8, and pH 6–7) harbored significantly different fungal communities. Lefse analysis using the factorial Kruskal–Wallis test showed that the many phylogenetic groups at order, family, and genus levels can be significantly distinguished among three soil types (i.e., pH 8–9, pH 7–8, and pH 6–7) (**Figures 3** and **4**). For example, at the order level, Verrucariales (*p* < 0.05) was found to predominate in soil samples of pH 8–9, whereas Sordariales (*p* < 0.05) predominated in soil samples of pH 7–8 and Coniochaetales (*p* < 0.05) predominated in soil samples of pH 6–7. At the genus level, *Rhinocladiella* (*p* < 0.01), and *Alta* (*p* < 0.01) were found to predominate in soils of pH 8–9, whereas *Nectria* (*p* < 0.05) and *Neonectria* (*p* < 0.05) were found to predominate in soils of pH 7–8 (Supplementary Table S3).

## Discussion

The aim of the present study was to analyze the soil fungal communities in the High Arctic tundra using high-throughput pyrosequencing. This study demonstrated a close relationship between soil fungal communities and certain soil properties in the Ny-Ålesund Region, which was not clarified before in this High Arctic region.

### Soil Fungal Communities in High Arctic Tundra are Highly Diverse

Despite the geographic isolation and High Arctic climate of Svalbard, the soil fungal communities are surprisingly diverse in this High Arctic region. Members of Ascomycota were more frequently identified in soils than those of Basidiomycota and Chytridiomycota. Additionally, Zygomycota, Glomeromycota, and Rozellomycota were less abundant in the Arctic soil environments. These findings were consistent with previous studies of soil fungi in Arctic tundra. Gittel et al. (2014) reported that the most abundant phyla were Ascomycota and Basidiomycota and other phyla (i.e., Chytridiomycota, Glomeromycota, and Zygomycota) accounted for only a minor fraction in Siberian tundra (High Arctic). Wallenstein et al. (2007) reported that soil fungal communities were dominated by Basidomycota and there were also a large proportion of Ascomycota, and lesser relative amounts of Zygomycota, Chytridmycota, and Glomeromycota in Alaska (Low Arctic).

The dominant fungal orders detected in this study were Helotiales (Leotiomycetes) in Ascomycota, and Agaricales (Agaricomycetes) in Basidiomycota. The order Helotiales was previously observed in plant rhizosphere in alpine and Arctic regions (Bjorbækmo et al., 2010; Walker et al., 2011). This order represents the largest and the most diverse group in the Leotiomycetes (Wang et al., 2006) and members of the order can function as mycorrhizae, ectomycorrhizal parasites, terrestrial saprobes, and root symbionts (Vrålstad et al., 2002). Deslippe et al. (2012) found that Ascomycota were dominated by Helotiales, and Basidomycota were dominatead by Agaricales in Alaska (Low Arctic). In Siberian tundra (High Arctic), Ascomycota were dominated by Leotimycetes, whereas Basidiomycota were dominated by Agaricomycetes (Gittel et al., 2014).

Among the 95 fungal genera detected in the present study, only 24 have previously been reported from soils in the Arctic regions, including *Acremonium, Alnicola, Alternaria, Aspergillus, Cladosporium, Clavulina, Cortinarius, Cylindrocarpon, Geomyces, Hebeloma, Inocybe, Laccaria, Mortierella, Neonectria, Phialocephala, Phoma, Preussia, Rhodotorula, Sebacina, Thelebolus, Tolypocladium, Tomentella, Trichosporon, Varicosporium* (Bergero et al., 1999; Kurek et al., 2007; Geml et al., 2012; Meyling et al., 2012; Singh et al., 2012; Ali et al., 2013; Gittel et al., 2014). Interestingly, the soil fungal genera detected in this study included ectomycorrhizal fungi (e.g., *Alnicola, Cortinarius, Hebeloma, Inocybe, Laccaria, Tomentella*), lichenized fungi (e.g., *Polyblasitia, Thelidium, Atla, Verrucaria, Stereocaulon, Lecidea, Rhizocarpon*), saprotrophic fungi (e.g., *Fusarium, Mortierella, Nectria*), dark septate root endophytes (e.g., *Phialocephala, Cadophora, Leptodontidium*), and yeasts (e.g., *Pichia, Cryptococcus, Dioszegia, Glaciozyma, Mrakia, Malassezia, Rhodotorula, Trichosporon*). Taken together, these data suggest that soil fungal communities found in this High Arctic region are highly diverse and these soil fungi are widely distributed and may play various roles in the Arctic soil environments.

### Soil pH is a Key Factor Determining the Soil Fungal Community Structure in High Arctic Tundra

In previous studies, researchers found that Arctic fungal communities in roots and soils could change in response to environmental factors, such as pH, temperature, precipitation, and nutrient availability (e.g., C/N ratio, P content) (Clemmensen et al., 2006; Fujimura and Egger, 2012; Timling et al., 2012). Both Robinson et al. (2004) and Deslippe et al. (2012) found Arctic fungal community structure differed between organic soils and mineral soils, with this variation between soils probably related to the higher C and other nutrient content in the organic soil.

From db-RDA analysis in this study, soil pH was the most important environmental factor (*p* = 0.001) that was correlated with soil fungal communities in the Ny-Ålesund Region. An ANOSIM analysis also revealed that fungal communities inhabiting soils of different pH levels were significantly different (*p* < 0.05). All these results suggested that soil pH was a key factor shaping the fungal community structure in this High Arctic region. Silicate silicon (*p* < 0.01), organic C (*p* < 0.05), and organic N (*p* < 0.05) were also found to be significantly related to soil fungal community composition in this study. It is generally believed that some soil characteristics (e.g., organic matter) are directly or indirectly related to soil pH levels. Interestingly, significant correlations were observed between pH and other soil properties in the present study, including soluble silicate (*r* = 0.768, *p* < 0.01), organic C (*r* = -0.596, *p* < 0.05), and organic N (*r* = -0.666, *p* < 0.05) (Supplementary Table S1). The organic carbon (C) and nitrogen (N) could be transferred into soils by vegetation and animal activities. Stempniewicz et al. (2007) reported that animal feces, carcasses and plants are important contributors to the nitrogen (N) and phosphorus (P) cycles in High Arctic soils. Liu et al. (2015) found that the vegetation had a significant effect on the content of soluble silicon in soils of Ny-Ålesund.

### Some Fungal Phylogenetic Groups Show Significantly Different Responses to Soil pH

Soil pH may directly affect fungal community composition by imposing a physiological constraint on fungal survival and growth and some fungal taxa may be unable to grow or survive if the soil pH falls outside a certain range. Fungi generally grow well in acidic conditions (Dix and Webster, 1995), but some fungi (e.g., *Mortierella, Peziza*) grow well in neutral to slightly alkaline conditions (Warcup, 1951; Yamanaka, 2003). As not all members of the same phylum behaved in the same way, it is interesting to examine the different responses of fungal phylogenetic groups, especially groups at the lower taxonomic levels. In this study, many phylogenetic groups at order, family, and genus levels showed significantly different responses to soil pH levels (as shown in **Figure 3**).

Interestingly, soil samples in glacier foreland (at high altitude) were generally alkaline (pH 8–9), whereas pH values of soil samples, which are far away from glacier forelands, were generally below 8. Glacial retreat leads to subsequent exposure of foreland soils to atmospheric conditions, thus creating a sequence of change in the soil environments, such as the low levels of nutrients present in foreland soils and the decrease in soil pH that occurs along the chronosequence (Hodkinson et al., 2003). We hypothesize those lichen-forming fungi, e.g., Verrucariales (order), Verrucariaceae (family), and *Atla* (genus), may serve as pioneer organisms and firstly colonize and dominate in the glacier foreland soils. A previous study also reported that some members of Verrucariaceae prefer sites with high pH (calcareous sites) (Shivarov, 2013; Gueidan et al., 2014). In addition, non-lichen forming fungi predominated in soil samples of pH < 8.0. For example, Sordariales (order) predominated in soil samples of pH 7–8, whereas Coniochaetales (order) predominated in soil samples of pH 6–7. Yamanaka (2003) found that many of the saprotrophic species grew well at pH 7 or 8 and the ectomycorrhizal species showed optimum growth at pH 5 or 6. Most members of Sordariales are saprobic and consist of mostly wood- and dung-inhabiting species, whereas members of Coniochaetales (order) always occur on wood, dung or soil (Zhang et al., 2006).

In summary, soil pH was a key factor in determining the soil fungal community composition in this High Arctic region. In addition, there are many unmeasured environmental factors, which may be related to the soil fungal community composition in the High Arctic tundra, such as vegetation composition and productivity. With a warming climate, an enhanced colonization of vascular plants would then affect soil fungal community structure and thus further experiments should be performed to confirm the influence of plant vegetation in the High Arctic tundra. The other question is as follows: what are the possible ecological roles and functions of these soil fungi in the Arctic tundra? Perhaps the use of other molecular tools (e.g., transcriptome analysis using next-generation sequencing) will allow us to clarify their ecological functions in Arctic tundra soils.

## Author Contributions

TZ planned the study, collected samples, conducted lab work, and wrote the manuscript; NW performed geochemical analyses of soil samples; HL and YZ conducted parts of lab work; LY contributed with planning the project and revising the manuscript. All authors reviewed the manuscript.

## Conflict of Interest Statement

The authors declare that the research was conducted in the absence of any commercial or financial relationships that could be construed as a potential conflict of interest.

## Footnotes

1 https://en.wikipedia.org/wiki/Ny-Ålesund#cite_note-77

2 https://unite.ut.ee/analysis.php

3 http://www.ncbi.nlm.nih.gov/genbank
