# Supplementary material for: Soil pH is a Key Determinant of Soil Fungal Community Composition in the Ny-Ålesund Region, Svalbard (High Arctic)
Source: Front Microbiol. 2016 Feb 26;7:227. doi: 10.3389/fmicb.2016.00227 (PMC4767930; doi:10.3389/fmicb.2016.00227)
Supplement: Supplementary file 1 [file Table_1.DOCX]

**Table S1 | Pearson correlation analysis of different environmental factors.**

|  | pH | p value |
| --- | --- | --- |
| Organic C | -0.596 | 0.043* |
| Organic N | -0.666 | 0.013* |
| NH_4_^+^-N | -0.209 | 0.492 |
| NO_2_^-^-N | 0.127 | 0.680 |
| NO_3_^-^-N | -0.204 | 0.504 |
| PO_4_^3-^-P | -0.315 | 0.294 |
| SiO_4_^2-^-Si | 0.768 | 0.002** |

*Correlation is significant at the 0.05 level (2-tailed).

** Correlation is significant at the 0.01 level (2-tailed).
