# Supplementary material for: Soil pH is a Key Determinant of Soil Fungal Community Composition in the Ny-Ålesund Region, Svalbard (High Arctic)
Source: Front Microbiol. 2016 Feb 26;7:227. doi: 10.3389/fmicb.2016.00227 (PMC4767930; doi:10.3389/fmicb.2016.00227)
Supplement: Supplementary file 2 [file Table_2.DOCX]

Table S2 | Information on the 541 OTUs found in the 13 soil samples.

| **ID** | **Freq.** | **Reads** | **phylum** | **Order** | **Family** | **Similarity** | **Accession** | **Close GenBank match** | **Originally reported habitat**^§^ |
| --- | --- | --- | --- | --- | --- | --- | --- | --- | --- |
| 1 | 2 | 2 | Ascomycota | Coniochaetales | Coniochaetaceae | 375/384(98%) | JQ346870 | Uncultured Sordariomycetes | Roots of herbs in alpine meadows in China |
|  |  |  |  |  |  | 377/404(93%) | KF285995 | *Coniochaeta ligniaria* | -- |
| 2 | 1 | 8 | Basidiomycota | Unassigned | Unassigned | 497/500(99%) | KC965856 | Uncultured fungus | Arctic soil in Canada (78.78N;103.55W) |
|  |  |  |  |  |  | 299/370(81%) | AM901823 | Uncultured Basidiomycota | -- |
| 3 | 3 | 4 | Ascomycota | Acarosporales | Acarosporaceae | 483/517(93%) | DQ534451 | *Acarospora austroshetlandica* | -- |
| 4 | 2 | 67 | Ascomycota | Capnodiales | Teratosphaeriaceae | 504/519(97%) | KC965669 | Uncultured fungus (Capnodiales) | Arctic soil in USA (69.15N;148.85W) |
|  |  |  |  |  |  | 498/544(92%) | JF499843 | *Penidiella ellipsoidea* | -- |
| 5 | 1 | 5 | Ascomycota | Helotiales | Unassigned | 234/244(96%) | DQ421214 | Uncultured fungus | Soil in USA (Cedar Creek, Minnesota) |
|  |  |  |  |  |  | 231/247(93%) | KC694156 | Uncultured *Tetracladium* | -- |
| 6 | 1 | 6 | Ascomycota | Helotiales | Unassigned | 500/504(99%) | KF274337 | Uncultured fungus | Wood stump in Finland |
|  |  |  |  |  |  | 498/504(99%) | HQ599580 | *Xenopolyscytalum pinea** | Needles of *Pinus* sp. in Netherlands |
| 7 | 1 | 18 | Basidiomycota | Sebacinales | Sebacinaceae | 477/479(99%) | KF000470 | Uncultured *Sebacina*^#^ | Ectomycorrhizal root tip in Germany |
| 8 | 2 | 49 | Ascomycota | Helotiales | Helotiaceae | 469/502(93%) | KC455359 | Uncultured Helotiaceae | -- |
| 9 | 4 | 23 | Ascomycota | Heloeiales | Leotiaceae | 502/505(99%) | KC966006 | Uncultured fungus | Arctic soil in Canada (78.78N;103.55W) |
|  |  |  |  |  |  | 484/510(95%) | KC834057 | *Gorgomyces honrubiae*^#^ | -- |
| 10 | 8 | 29 | Ascomycota | Helotiales | Helotiaceae | 472/479(99%) | HQ850136 | Uncultured fungus | Plant root hairs in China |
|  |  |  |  |  |  | 471/479(98%) | KP234354 | *Fontanospora eccentrica*^#^ | Chile: Patagonia |
| 11 | 1 | 21 | Basidiomycota | Leucosporidiales | unassigned | 387/430(90%) | KM504475 | Uncultured fungus | -- |
|  |  |  |  |  |  | 371/405(92%) | AB474394 | *Rhodotorula* sp. | -- |
| 12 | 1 | 6 | Unassigned | Unassigned | Unassigned | 290/349(83%) | FJ626913 | Uncultured fungus | -- |
| 13 | 4 | 14 | Basidiomycota | Kriegeriales | Kriegeriaceae | 468/473(99%) | KC333169 | *Rhodotorula psychrophenolica** | Cryoconite sediment in Svalbard |
| 14 | 2 | 7 | Basidiomycota | Filobasidiales | Unassigned | 350/351(99%) | KC965257 | Uncultured fungus | Arctic soil in USA (70.31N;147.99W) |
|  |  |  |  |  |  | 338/338(100%) | AB032676 | *Cryptococcus gastricus** | Unreported |
| 15 | 1 | 4 | Basidiomycota | Tremellales | Unassigned | 350/351(99%) | AB222614 | Uncultured fungus | Italian ryegrass seed |
|  |  |  |  |  |  | 336/337(99%) | AJ581066 | *Dioszegia crocea** | Plant roots in Germany |
| 16 | 2 | 30 | Ascomycota | Helotiales | Helotiaceae | 461/466(99%) | AJ430395 | *Hymenoscyphus rhodoleucus** | Plant root in Norway |
| 17 | 1 | 60 | Unassigned | Unassigned | Unassigned | 431/545(79%) | HE977533 | Uncultured soil fungus | -- |
| 18 | 5 | 9 | Zygomycota | Mortierellales | Mortierellaceae | 338/340(99%) | HM240104 | Uncultured fungus | Grassland soil in USA |
|  |  |  |  |  |  | 328/329(99%) | JX270348 | *Mortierella* sp. ^#^ | Soil in USA: New Hampshire |
| 19 | 1 | 8 | Unassigned | Unassigned | Unassigned | 185/218(85%) | AM260867 | Uncultured fungus | -- |
| 20 | 8 | 132 | Ascomycota | Pleosporales | Didymellaceae | 520/523(99%) | AY337712 | *Phoma herbarum** | Chinook salmon |
| 21 | 3 | 38 | Ascomycota | Helotiales | Unassigend | 453/514(88%) | KM113762 | Helotiales sp. | -- |
| 22 | 3 | 70 | Ascomycota | Pleosporales | Leptosphaeriaceae | 501/516(97%) | JX489835 | Uncultured soil fungus | Soil in China: Heilongjiang, Harbin |
|  |  |  |  |  |  | 442/445(99%) | HM589500 | *Leptosphaeria sclerotioides** | Plant in Canada: Saskatchewan |
| 23 | 1 | 8 | Ascomycota | Sordariales | Chaetomiaceae | 352/368(96%) | GU174422 | Uncultured fungus | -- |
|  |  |  |  |  |  | 339/356(95%) | AM292049 | *Trichocladium opacum*^#^ | -- |
| 24 | 6 | 21 | Ascomycota | Pleosporales | Didymosphaeriaceae | 510/516(99%) | GU174360 | Uncultured fungus | Forest floor in USA: Michigan |
|  |  |  |  |  |  | 510/516(99%) | AB665314 | *Coniothyrium fuckelii** | Volcanic ash soil in Japan |
| 25 | 2 | 100 | Ascomycota | Capnodiales | Teratosphaeriaceae | 509/510(99%) | FJ553032 | Uncultured Dothideomycetidae | Forest soil in Canada |
|  |  |  |  |  |  | 464/517(90%) | EU707864 | *Teratosphaeria jonkershoekensis* | -- |
| 26 | 8 | 804 | Ascomycota | Chaetothyriales | Herpotrichiellaceae | 565/579(98%) | KC965227 | Uncultured fungus | Arctic soil in Canada (76.23 N 119.30 W) |
|  |  |  |  |  |  | 503/517(97%) | FJ948175 | *Rhinocladiella* sp. ^#^ | Rock in China: Xinjiang |
| 27 | 1 | 12 | Ascomycota | Verrucariales | Verrucariaceae | 312/316(99%) | EU697640 | *Sporodictyon schaererianum** | Northern Europe and the adjacent Arctic |
| 28 | 6 | 63 | Ascomycota | Verrucariales | Unassigend | 341/357(96%) | KC966180 | Uncultured fungus | Arctic soil in USA (69.67 N 148.72 W) |
|  |  |  |  |  |  | 319/360(89%) | FJ645264 | *Polyblastia* sp. | -- |
| 29 | 2 | 4 | Basidiomycota | Microbotryales | Microbotryaceae | 279/335(83%) | JQ272411 | *Leucosporidium* sp. | -- |
| 30 | 2 | 87 | Ascomycota | Capnodiales | Unassigned | 527/528(99%) | KC966349 | Uncultured fungus | Arctic soil in Canada (76.23 N 119.30 W) |
|  |  |  |  |  |  | 492/497(99%) | FJ415475 | *Elasticomyces elasticus* * | Antarctica |
| 31 | 1 | 5 | Unassigned | Unassigned | Unassigned | 344/403(85%) | KP889925 | Uncultured fungus | -- |
| 32 | 4 | 15 | Ascomycota | Chaetothyriales | Herpotrichiellaceae | 570/580(98%) | FJ554179 | Uncultured Herpotrichiellaceae | Forest soil in Canada |
|  |  |  |  |  |  | 546/587(93%) | EU139138 | *Rhinocladiella* sp. | -- |
| 33 | 2 | 133 | Basidiomycota | Thelephorales | Thelephoraceae | 482/482(100%) | KF000627 | Uncultured Thelephorales | Ectomycorrhizal root tip in Germany |
|  |  |  |  |  |  | 482/494(97%) | JQ711817 | *Tomentella* sp. ^#^ | Ectomycorrhizam in Canada: BC (55.45 N 123.20 W) |
| 34 | 1 | 27 | Zygomycota | Mortierellales | Mortierellaceae | 403/417(97%) | EU292438 | Uncultured fungus | Soil in USA : Bonanza Creek Experimental Forest |
|  |  |  |  |  |  | 402/426(94%) | JX270427 | *Mortierella* sp. | -- |
| 35 | 4 | 147 | Basidiomycota | Agaricales | Cortinariaceae | 400/403(99%) | GU817098 | Uncultured fungus | Plant root in Svalbard Midtre Lovenbreen (78.90N 12.08E) |
|  |  |  |  |  |  | 399/403(99%) | EU668257 | Uncultured *Cortinarius* ^#^ | Plant roots in forest soi in Wales |
| 36 | 1 | 6 | Ascomycota | Helotiales | Unassigned | 245/261(94%) | KM113762 | Helotiales sp. | -- |
| 37 | 1 | 15 | Ascomycota | Capnodiales | Unassigned | 326/373(87%) | GQ852682 | *Teratosphaeria cryptica* | -- |
| 38 | 3 | 36 | Unassigned | Unassigned | Unassigned | 346/367(94%) | KP889857 | Uncultured fungus | -- |
| 39 | 1 | 27 | Ascomycota | Lecanorales | Unassigned | 394/464(85%) | HM161487 | *Umbilicaria haplocarpa* | -- |
| 40 | 3 | 38 | Glomeromycota | Unassigned | Unassigned | 292/310(94%) | JX898577 | Uncultured Glomeromycota | -- |
| 41 | 2 | 17 | Basidiomycota | Agaricales | Cortinariaceae | 489/495(99%) | AB669647 | Uncultured mycorrhizal fungus | Mycorrhizal root tip in Japan (43.54 N 143.16 E) |
|  |  |  |  |  |  | 448/453(99%) | FJ039560 | *Cortinarius cf. flos-paludis** | Canada |
| 42 | 2 | 88 | Ascomycota | Lecanorales | Stereocaulaceae | 553/566(98%) | FJ552738 | Uncultured Stereocaulaceae | Forest soil in Canada |
|  |  |  |  |  |  | 547/567(97%) | DQ534486 | *Stereocaulon alpinum* ^#^ | Antarctica |
| 43 | 5 | 453 | Ascomycota | Lecideales | Lecideaceae | 477/533(89%) | KC965547 | Uncultured fungus | -- |
|  |  |  |  |  |  | 423/464(91%) | AM292669 | *Bilimbia microcarpa* | -- |
| 44 | 2 | 15 | Ascomycota | Helotiales | Dermateaceae | 503/513(98%) | KF800215 | Uncultured fungus | Indoor air in USA: Missouri, Kansas City |
|  |  |  |  |  |  | 501/513(98%) | KC753443 | Uncultured *Oculimacula* ^#^ | Stem base of plant in Sweden |
| 45 | 8 | 467 | Ascomycota | Thelebolales | Thelebolaceae | 545/547(99%) | JX489808 | Uncultured soil fungus | Soil in China: Heilongjiang, Harbin |
|  |  |  |  |  |  | 524/526(99%) | JX171196 | *Thelebolus globosus** | Antarctica |
| 46 | 2 | 3 | Zygomycota | Mortierellales | Mortierellaceae | 364/367(99%) | KC965787 | Uncultured fungus | Arctic soil in Canada (76.23 N 119.30 W) |
|  |  |  |  |  |  | 360/394(91%) | KC922119 | Uncultured *Mortierella* | -- |
| 47 | 1 | 3 | Ascomycota | Chaetothyriales | Herpotrichiellaceae | 379/417(91%) | KC965460 | Uncultured fungus | -- |
|  |  |  |  |  |  | 376/420(90%) | EU139138 | *Rhinocladiella* sp. | -- |
| 48 | 8 | 206 | Ascomycotaa | Venturiales | Venturiaceae | 551/552(99%) | AB916509 | *Venturia* sp. ^#^ | Bird feather in Norway |
|  |  |  |  |  |  | 549/553(99%) | KC965144 | Uncultured fungus | Arctic soil in Canada (76.23 N 119.30 W) |
| 49 | 2 | 10 | Ascomycota | Verrucariales | Verrucariaceae | 418/438(95%) | FJ664852 | *Verrucaria* sp. ^#^ | -- |
|  |  |  |  |  |  | 408/417(97%) | KC965347 | Uncultured fungus | Arctic soil in Canada (78.78 N 103.55 w |
| 50 | 1 | 19 | Ascomycota | Helotiales | Unassigned | 517/523(99%) | KF296739 | Uncultured fungus | Arctic soil in Canada (76.23 N 119.30 W) |
|  |  |  |  |  |  | 448/505(89%) | JX630692 | Uncultured *Tetracladium* | -- |
| 51 | 8 | 1574 | Ascomycota | Verrucariales | Verrucariaceae | 482/502(96%) | KC966180 | Uncultured fungus | Arctic soil in USA (69.67 N 148.72 W) |
|  |  |  |  |  |  | 427/472(90%) | EU559739 | *Polyblastia* sp. | -- |
| 52 | 1 | 3 | Ascomycota | Helotiales | Unassigned | 488/503(97%) | KC965466 | Uncultured fungus | Arctic soil in Canada (73.22 N 119.56 W) |
|  |  |  |  |  |  | 492/526(94%) | FJ378851 | Uncultured Helotiales isolate | -- |
| 53 | 1 | 282 | Basidiomycota | Agaricales | Tricholomataceae | 462/476(97%) | KC966276 | Uncultured fungus | Arctic soil in USA (69.67 N 148.72 W) |
|  |  |  |  |  |  | 454/479(95%) | JF519051 | Uncultured Tricholomataceae | Plant root in Austria (48.117 N 16.050 E) |
| 54 | 1 | 3 | Glomeromycota | Unassigned | Unassigned | 204/228(89%) | KP889758 | Uncultured fungus | -- |
|  |  |  |  |  |  | 178/204(87%) | EF619907 | Uncultured Glomeromycota | -- |
| 55 | 1 | 4 | Ascomycota | Pleosporales | Melanommataceae | 434/471(92%) | FN397293 | Uncultured fungus | -- |
|  |  |  |  |  |  | 424/472(90%) | KF646104 | *Tumularia* sp. | -- |
| 56 | 3 | 370 | Ascomycota | Helotiales | Unassigned | 448/476(94%) | KC455359 | Uncultured Helotiales | -- |
| 57 | 5 | 178 | Basidiomycota | Sebacinales | Sebacinaceae | 341/356(96%) | KC966180 | Uncultured fungus | Arctic soil in USA (69.67 N 148.72 W) |
|  |  |  |  |  |  | 286/306(93%) | GQ907144 | Uncultured *Sebacina* | -- |
| 58 | 1 | 160 | Ascomycota | Helotiales | Unassigned | 496/514(96%) | KC965355 | Uncultured fungus | Arctic soil in Canada (78.78 N 103.55 W) |
|  |  |  |  |  |  | 433/470(94%) | AY465452 | Helotiales sp. | -- |
| 59 | 1 | 138 | Chytridiomycota | Unassigned | Unassigned | 412/466(88%) | KC965876 | Uncultured fungus | -- |
|  |  |  |  |  |  | 296/355(83%) | HQ191313 | Uncultured Chytridiomycota | -- |
| 60 | 1 | 8 | Basidiomycota | Agaricales | Cortinariaceae | 495/498(99%) | GU817146 | Uncultured fungus | Plant root in Svalbard (78.90N, 12.08E) |
|  |  |  |  |  |  | 474/478(99%) | JF304378 | Uncultured *Cortinarius* ^#^ | Soil in Svalbard |
| 61 | 1 | 4 | Ascomycota | Unassigned | Unassigned | 238/250(95%) | KF617525 | Uncultured fungus | Forest soil in USA: Alaska (65.1617 N 147.4879 W) |
|  |  |  |  |  |  | 205/216(95%) | KF730837 | *Triscelophorus cf. acuminatus*^#^ | Unreported |
| 62 | 6 | 40 | Ascomycota | Pleosporales | Sporomiaceae | 514/516(99%) | JQ666535 | Uncultured soil fungus | Forest soil in China: Changbai mountain |
|  |  |  |  |  |  | 499/505(99%) | FJ903358 | *Preussia* sp. ^#^ | Decayed wood in Latvia |
| 63 | 2 | 3 | Basidiomycota | Agaricales | Tricholomataceae | 427/443(96%) | KF296734 | Uncultured fungus | Arctic soil in Canada (76.23 N 119.30 W) |
|  |  |  |  |  |  | 349/389(90%) | JF908757 | *Arrhenia elegans* | -- |
| 64 | 1 | 62 | Unassigned | Unassigned | Unassigned | 376/416(90%) | KC966235 | Uncultured fungus | -- |
| 65 | 1 | 33 | Ascomycota | Chaetothyriales | Herpotrichiellaceae | 495/514(96%) | KC966346 | Uncultured fungus | Arctic soil in Canada (76.23N;119.30W) |
|  |  |  |  |  |  | 438/477(92%) | FJ265748 | *Cladophialophora* sp. | -- |
| 66 | 1 | 4 | Ascomycota | Hypocreales | Nectriaceae | 213/218(98%) | JX325673 | Uncultured fungus | Soil in USA |
|  |  |  |  |  |  | 212/218(97%) | KM248576 | *Cylindrocarpon* sp. ^#^ | Plant germinants in Austria (47.75N, 15.06E) |
| 67 | 1 | 18 | Ascomycota | Helotiales | Helotiaceae | 438/491(89%) | KP889799 | Uncultured fungus | -- |
|  |  |  |  |  |  | 432/483(89%) | FJ440900 | Uncultured Helotiaceae | -- |
| 68 | 6 | 52 | Ascomycota | Verrucariales | Verrucariaceae | 364/370(98%) | KC965643 | Uncultured fungus | Arctic soil in Canada (76.23N, 119.30W) |
|  |  |  |  |  |  | 390/409(95%) | FJ664852 | *Verrucaria* sp. ^#^ | -- |
| 69 | 1 | 6 | Ascomycota | Coniochaetales | Coniochaetaceae | 232/253(92%) | JQ760239 | Sordariomycetes sp. | -- |
|  |  |  |  |  |  | 235/261(90%) | KP235712 | Uncultured *Coniochaeta* | -- |
| 70 | 1 | 26 | Glomeromycota | Unassigned | Unassigned | 452/456(99%) | KF297176 | Uncultured fungus | Arctic soil in Canada (76.23 N 119.30W) |
|  |  |  |  |  |  | 397/417(95%) | EF619906 | Uncultured Glomeromycota | -- |
| 71 | 8 | 247 | Ascomycota | Helotiales | Unassigned | 434/470(92%) | KC965311 | Uncultured fungus | -- |
|  |  |  |  |  |  | 463/527(88%) | JN859274 | Helotiales sp. | -- |
| 72 | 2 | 51 | Basidiomycota | Tremellales | Unassigned | 475/478(99%) | JQ857039 | *Dioszegia fristingensis* * | King George Island, Antarctica |
| 73 | 2 | 2 | Ascomycota | Unassigned | Unassigned | 256/303(84%) | KC965399 | Uncultured fungus | -- |
|  |  |  |  |  |  | 252/308(82%) | AJ971444 | *Coniosporium* sp. | -- |
| 74 | 4 | 10 | Ascomycota | Helotiales | Hyaloscyphaceae | 511/515(99%) | KC965972 | Uncultured fungus | Arctic soil in USA (69.15 N 148.85 W) |
|  |  |  |  |  |  | 517/538(97%) | HQ212216 | Uncultured *Clathrosphaerina*^#^ | Arctic soil |
| 75 | 1 | 18 | Ascomycota | Helotiales | Unassigned | 493/505(98%) | FJ554233 | Uncultured Helotiales | Forest soil in Canada |
|  |  |  |  |  |  | 479/492(97%) | EF093148 | Helotiales sp. | Plant root tip in Cezch |
| 76 | 5 | 27 | Basidiomycota | Agaricales | Tricholomataceae | 479/482(99%) | GU817186 | Uncultured fungus | Plant root in Svalbard (78.90 N 12.08 E) |
|  |  |  |  |  |  | 477/482(99%) | JX504099 | *Laccaria aff. montana** | China: Tibet |
| 77 | 3 | 361 | Ascomycota | Lecanorales | Porpidiaceae | 428/440(97%) | KC965547 | Uncultured fungus | Arctic soil in Canada (76.23N; 119.30W) |
|  |  |  |  |  |  | 423/435(97%) | AJ247567 | *Biatora carneoalbida*^#^ | Finland: Tavastia australis |
| 78 | 6 | 132 | Unassigned | Unassigned | Unassigned | 252/319(79%) | JX363695 | Uncultured fungus | -- |
| 79 | 1 | 4 | Basidiomycota | Agaricales | Inocybaceae | 416/417(99%) | HQ604595 | *Inocybe xanthomelas** | Canada |
| 80 | 1 | 3 | Ascomycota | Pleosporales | Leptosphaeriaceae | 397/397(100%) | KF297170 | Uncultured fungus | Arctic soil in Canada (78.78 N 103.55 W) |
|  |  |  |  |  |  | 382/383(99%) | FJ179159 | *Leptosphaeria sclerotioides** | Unreported |
| 81 | 5 | 224 | Ascomycota | Helotiales | Unassigned | 392/404(97%) | HM069356 | Uncultured fungus | Pine forest soil in Finland |
|  |  |  |  |  |  | 384/393(97%) | HQ211545 | Uncultured Helotiales | Arctic soil |
| 82 | 2 | 81 | Glomeromycota | Unassigned | Unassigned | 446/457(98%) | KF296911 | Uncultured fungus | Arctic soil in Canada (73.22 N 119.56 W) |
|  |  |  |  |  |  | 337/386(87%) | GU392007 | Uncultured Glomeromycota | -- |
| 83 | 5 | 70 | Ascomycota | Sordariales | Lasiosphaeriaceae | 492/510(96%) | EU490132 | Uncultured soil fungus | -- |
|  |  |  |  |  |  | 491/510(96%) | KC694149 | Uncultured Lasiosphaeriaceae | Plant roots in Sweden |
| 84 | 1 | 10 | Unassigned | Unassigned | Unassigned | 380/438(87%) | HE977533 | Uncultured soil fungus | -- |
| 85 | 6 | 649 | Ascomycota | Helotiales | Unassigned | 407/426(96%) | FJ554141 | Uncultured Sordariomycetes | -- |
|  |  |  |  |  |  | 209/209(100%) | AJ430214 | *Phialocephala fortinii** | Ectomycorrhizal root |
| 86 | 1 | 11 | Ascomycota | Helotiales | Unassigned | 310/323(96%) | KC965311 | Uncultured fungus | Arctic soil in Canada (73.22 N 119.56 W) |
|  |  |  |  |  |  | 315/345(91%) | GU327472 | Uncultured *Tetracladium* | -- |
| 87 | 2 | 6 | Zygomycota | Mortierellales | Mortierellaceae | 366/374(98%) | KM889556 | Uncultured *Mortierella*^#^ | Farm soil in USA: Illinois |
| 88 | 1 | 4 | Glomeromycota | Unassigned | Unassigned | 412/453(91%) | DQ421311 | Uncultured soil fungus | -- |
|  |  |  |  |  |  | 228/285(80%) | AM713402 | *Diversispora* sp. | -- |
| 89 | 3 | 55 | Ascomycota | Peltigerales | Lobariaceae | 461/525(88%) | KF296938 | Uncultured fungus | -- |
|  |  |  |  |  |  | 211/223(94%) | EU558725 | *Pseudocyphellaria endochrysea* | -- |
| 90 | 1 | 7 | Ascomycota | Capnodiales | Unassigned | 385/404(95%) | KC965273 | Uncultured fungus | Arctic soil in Canada (78.78 N 103.55 W) |
|  |  |  |  |  |  | 363/383(95%) | JF691102 | Uncultured Capnodiales | Plant root in Reunion |
| 91 | 1 | 6 | Ascomycota | Unassigned | Unassigned | 343/380(90%) | KF296911 | Uncultured fungus | -- |
|  |  |  |  |  |  | 240/251(96%) | JX675145 | Uncultured Ascomycota | -- |
| 92 | 1 | 2 | Basidiomycota | Tremellales | Unassigned | 407/408(99%) | DQ000318 | *Cryptococcus tephrensis** | Plant leaves |
| 93 | 1 | 5 | Basidiomycota | Agaricales | Inocybaceae | 457/459(99%) | KC965598 | Uncultured fungus | Arctic soil in USA (69.67 N 148.72 W) |
|  |  |  |  |  |  | 440/442(99%) | JF304339 | Uncultured *Inocybe*^#^ | Soil in Svalbard |
| 94 | 5 | 27 | Ascomycota | Helotiales | Unassigned | 347/348(99%) | KF000642 | Uncultured Helotiales | ectomycorrhizal root tip in Germany |
|  |  |  |  |  |  | 346/348(99%) | KC180685 | *Leptodontidium cf. orchidicola** | Plant root in Argentina |
| 95 | 4 | 24 | Ascomycota | Helotiales | Dermateaceae | 523/538(97%) | FN610995 | Uncultured fungus | sandy clayey soil in France (47.18 N 4.04 E) |
|  |  |  |  |  |  | 513/539(95%) | FJ553813 | Uncultured Dermateaceae | -- |
| 96 | 2 | 555 | Basidiomycota | Atheliales | Atheliaceae | 473/484(98%) | KC965449 | Uncultured fungus | Arctic soil in Canada (76.23 N 119.30W) |
|  |  |  |  |  |  | 367/399(92%) | EU118605 | *Athelia pyriformis* | -- |
| 97 | 1 | 2 | Ascomycota | Hypocreales | Bionectriaceae | 241/242(99%) | KC966181 | Uncultured fungus | Arctic soil in USA (69.67 N 148.72 W) |
|  |  |  |  |  |  | 227/257(88%) | AB540553 | *Gliomastix masseei* | -- |
| 98 | 2 | 9 | Basidiomycota | Unassigned | Unassigned | 388/413(94%) | KF296911 | Uncultured fungus | -- |
|  |  |  |  |  |  | 383/454(84%) | KF359632 | Basidiomycota sp. | -- |
| 99 | 2 | 153 | Ascomycota | Pezizales | Unassigned | 439/497(88%) | KC966365 | Uncultured fungus | -- |
|  |  |  |  |  |  | 443/511(87%) | FM206478 | *Tarzetta catinus* |  |
| 100 | 1 | 15 | Ascomycota | Unassigned | Unassigned | 232/292(79%) | AY425632 | *Protoblastenia incrustans* | -- |
| 101 | 1 | 3 | Basidiomycota | Agaricales | Cortinariaceae | 437/448(98%) | KC965299 | Uncultured fungus |  |
|  |  |  |  |  |  | 417/465(90%) | KP171143 | *Inocybe* sp. | -- |
| 102 | 1 | 41 | Basidiomycota | Agariostilbales | Unassigned | 511/519(98%) | KM504434 | Uncultured fungus | Ectomycorrhizae in subalpine soil (Austria: Haggen) |
|  |  |  |  |  |  | 375/426(88%) | KC836087 | *Kurtzmanomyces shapotouensis* | -- |
| 103 | 3 | 2032 | Ascomycota | Lecanorales | Unassigned | 491/516(95%) | KF297149 | Uncultured fungus | Arctic soil in Canada (76.23 N 119.30 W) |
|  |  |  |  |  |  | 437/493(89%) | EU401769 | *Melanelia sorediella* | -- |
| 104 | 1 | 30 | Basidiomycota | Agaricales | Cortinariaceae | 491/492(99%) | JX630354 | Uncultured *Inocybe*^#^ | Root system in USA (70.30 N 147.98 W) |
| 105 | 4 | 234 | Ascomycota | Coniochaetales | Coniochaetaceae | 414/421(98%) | FJ554141 | Uncultured Sordariomycetes | Forest soil in Canada |
|  |  |  |  |  |  | 401/427(94%) | KR935844 | *Coniochaeta ligniaria* | -- |
| 106 | 1 | 11 | Ascomycota | Helotiales | Helotiaceae | 420/420(100%) | KC965289 | Uncultured fungus | Arctic soil in Canada (76.23 N 119.30 W) |
|  |  |  |  |  |  | 396/438(90%) | FJ440900 | Uncultured Helotiaceae | -- |
| 107 | 1 | 48 | Basidiomycota | Tremellales | Unassigned | 454/456(99%) | AB476491 | Uncultured fungus | Finest root fragment of Vaccinium in Sweden |
|  |  |  |  |  |  | 435/495(88%) | HQ890367 | *Cryptococcus* sp. | -- |
| 108 | 3 | 7 | Ascomycota | Coniochaetales | Coniochaetaceae | 479/503(95%) | KJ957775 | *Coniochaeta* sp. ^#^ | -- |
|  |  |  |  |  |  | 310/312(99%) | JQ692166 | *Lecythophora* sp. ^#^ | Arctic soil |
| 109 | 1 | 9 | Ascomycota | Chaetothyriales | Herpotrichiellaceae | 437/445(98%) | KC966029 | Uncultured fungus | Arctic soil in Canada (76.23 N 119.30 W) |
|  |  |  |  |  |  | 427/470(91%) | FN555433 | Uncultured Ascomycota (Herpotrichiellaceae) | -- |
| 110 | 1 | 27 | Ascomycota | Pezizales | Pyronemataceae | 411/422(97%) | KJ754182 | Uncultured fungus | Ectomycorrhizal soil in Canada (54.65 N 118.98 W) |
|  |  |  |  |  |  | 410/422(97%) | GU452518 | Uncultured Pyronemataceae | Mycorrhizal root tip in Canada: British Columbia |
| 111 | 1 | 12 | Ascomycota | Pezizales | Pyronemataceae | 379/386(98%) | JN704831 | Uncultured *Pulvinula*^#^ | Mycorrhizal root tip in Mexico |
|  |  |  |  |  |  | 360/362(99%) | KC965492 | Uncultured fungus | Arctic soil in USA (69.67 N 148.72 W) |
| 112 | 2 | 90 | Ascomycota | Pleosporales | Unassigned | 512/514(99%) | KC966327 | Uncultured fungus | Arctic soil in Canada (78.78 N 103.55 W) |
|  |  |  |  |  |  | 464/474(98%) | FJ911882 | *Mycocentrospora* sp. ^#^ | Plant leaves in Antarctica |
| 113 | 3 | 21 | Zygomycota | Mortierellales | Mortierellaceae | 406/416(98%) | JQ666562 | Uncultured soil fungus | Forest soil in China: Changbai mountain |
|  |  |  |  |  |  | 400/414(97%) | KM889556 | Uncultured *Mortierella*^#^ | Farm soil in USA: Illinois |
| 114 | 1 | 10 | Chytridiomycota | Rhizophydiales | Rhizophydiaceae | 355/359(99%) | KF296852 | Uncultured fungus | Arctic soil in Canada (76.23 N 119.30 W) |
|  |  |  |  |  |  | 192/208(92%) | DQ485612 | *Rhizophydium carpophilum* | -- |
| 115 | 2 | 13 | Basidiomycota | Tremellales | Tremellaceae | 290/298(97%) | AM999701 | Uncultured fungus | Bryophyte in Norway: Telemark |
|  |  |  |  |  |  | 329/374(88%) | HQ631056 | Tremellaceae sp. | -- |
| 116 | 1 | 6 | Basidiomycota | Agaricales | Inocybaceae | 208/209(99%) | AM882947 | *Inocybe calamistrata** | Norway |
| 117 | 3 | 284 | Basidiomycota | Sebacinales | Sebacinaceae | 452/489(92%) | KC986274 | Uncultured *Sebacina* | -- |
| 118 | 1 | 9 | Ascomycota | Sordariales | Unassigned | 344/354(97%) | HM123260 | Fungal sp. | Lichen thallus in USA: Arizona (31.88 N 109.20 W) |
|  |  |  |  |  |  | 376/401(94%) | JN704834 | Uncultured Sordariales | -- |
| 119 | 1 | 53 | Ascomycota | Capnodiales | Unassigned | 340/389(87%) | EU707860 | *Teratosphaeria bellula* | -- |
| 120 | 1 | 8 | Ascomycota | Unassigned | Unassigned | 352/421(84%) | JX338972 | Uncultured fungus | -- |
|  |  |  |  |  |  | 362/441(82%) | JQ318662 | Uncultured Helotiales | -- |
| 121 | 3 | 21 | Basidiomycota | Sebacinales | Sebacinaceae | 457/457(100%) | KF636400 | Sebacinales sp. | Antarctica (60.72 S 45.63 W) |
|  |  |  |  |  |  | 494/499(99%) | GQ907097 | Uncultured *Sebacina*^#^ | Liverwort in United Kingdom: Scotland |
| 122 | 1 | 39 | Ascomycota | Verrucariales | Verrucariaceae | 486/506(96%) | KC966146 | Uncultured fungus | -- |
|  |  |  |  |  |  | 452/504(90%) | KM243199 | *Verrucaria humida* | -- |
| 123 | 1 | 12 | Ascomycota | Pezizales | Pyronemataceae | 509/524(97%) | JX630433 | Uncultured *Geopora*^#^ | Root system in Greenland: Thule (76.53 N 68.76 W) |
| 124 | 1 | 24 | Ascomycota | Helotiales | Unassigned | 474/486(98%) | KF617316 | Uncultured fungus | Forest soil in USA: Alaska (64.7657 N 148.2955 W) |
|  |  |  |  |  |  | 429/473(91%) | JQ346944 | Uncultured Helotiales | -- |
| 125 | 2 | 109 | Ascomycota | Pezizales | Pyronemataceae | 258/260(99%) | KC965444 | Uncultured fungus | Arctic soil in Canada (76.23 N 119.30 W) |
|  |  |  |  |  |  | 259/280(93%) | FM206430 | *Geopora* sp. | -- |
| 126 | 1 | 439 | Ascomycota | Hypocreales | Unassigned | 463/529(88%) | KF742558 | Uncultured fungus | -- |
|  |  |  |  |  |  | 453/521(87%) | KM248554 | *Ilyonectria* sp. | -- |
| 127 | 1 | 8 | Ascomycota | Verrucariales | Verrucariaceae | 512/539(95%) | HM239990 | Uncultured Ascomycota | -- |
|  |  |  |  |  |  | 477/499(96%) | FJ664859 | *Verrucaria* sp. ^#^ | -- |
| 128 | 4 | 74 | Basidiomycota | Agaricales | Cortinariaceae | 495/496(99%) | GU817125 | Uncultured fungus | Plant root in Svalbard (78.90 N 12.08 E) |
|  |  |  |  |  |  | 494/496 (99%) | JQ724019 | *Cortinarius atrocoeruleus** | Ectomycorrhiza in Sweden (59.49 N 17.40 E) |
| 129 | 8 | 272 | Ascomycota | Helotiales | Unassigned | 486/498(98%) | KC694156 | Uncultured *Tetracladium*^#^ | Sweden |
| 130 | 1 | 3 | Glomeromycota | Unassigned | Unassigned | 331/405(82%) | HQ257445 | Uncultured fungus | -- |
|  |  |  |  |  |  | 195/220(89%) | EF619903 | Uncultured Glomeromycota | -- |
| 131 | 3 | 86 | Basidiomycota | Tremellales | Tremellaceae | 478/480(99%) | JQ768931 | *Dioszegia fristingensis** | Glacier surface snow in China: Tibetan plateau |
| 132 | 1 | 8 | Basidiomycota | Agaricales | Inocybaceae | 415/464(89%) | JF908159 | *Inocybe aeruginascens* | -- |
| 133 | 2 | 21 | Ascomycota | Verrucariales | Verrucariaceae | 374/393(95%) | KC966146 | Uncultured fungus | -- |
|  |  |  |  |  |  | 386/435(89%) | FJ664852 | *Verrucaria* sp. | -- |
| 134 | 1 | 1450 | Ascomycota | Helotiales | Unassigned | 462/529(87%) | KJ396077 | *Neofabraea alba* | -- |
| 135 | 1 | 13 | Ascomycota | Chaetothyriales | Unassigned | 435/447(97%) | KF296926 | Uncultured fungus | Arctic soil in USA (69.67 N 148.72 W) |
|  |  |  |  |  |  | 394/430(92%) | KC243968 | Uncultured Chaetothyriales | -- |
| 136 | 5 | 24 | Ascomycota | Pezizales | Unassigned | 554/562(99%) | KT581903 | Uncultured fungus | Plant litter in Mexico |
|  |  |  |  |  |  | 554/562(99%) | GU931770 | Uncultured Pezizales | House dust in Canada |
| 137 | 2 | 4 | Unassigned | Unassigned | Unassigned | 266/335(79%) | JF439198 | Fungal sp. | -- |
| 138 | 1 | 13 | Chytridiomycota | Unassigned | Unassigned | 280/340(82%) | JX388918 | Uncultured fungus | -- |
|  |  |  |  |  |  | 151/161(94%) | DQ536497 | *Rhizophydium* sp. | -- |
| 139 | 2 | 5 | Ascomycota | Helotiales | Leotiaceae | 447/464(96%) | JF519259 | Uncultured *Alatospora*^#^ | -- |
| 140 | 1 | 18 | Glomeromycota | Unassigned | Unassigned | 486/497(98%) | EF521235 | Uncultured fungus | Spruce forest |
|  |  |  |  |  |  | 410/434(94%) | EF619904 | Uncultured Glomeromycota | -- |
| 141 | 5 | 108 | Ascomycota | Verrucariales | Unassigned | 500/506(99%) | KC965506 | Uncultured fungus | Arctic soil in USA (69.67 N 148.72 W) |
|  |  |  |  |  |  | 462/521(89%) | FJ664854 | *Verrucaria* sp. | -- |
| 142 | 5 | 38 | Ascomycota | Capnodiales | Cladosporiaceae | 558/565(99%) | AF393719 | *Cladosporium nigrellum** | Unreported |
| 143 | 4 | 76 | Glomeromycota | Unassigned | Unassigned | 295/339(87%) | JX365841 | Uncultured fungus | -- |
|  |  |  |  |  |  | 201/220(91%) | JX898577 | Uncultured Glomeromycota | -- |
| 144 | 3 | 180 | Basidiomycota | Agaricales | Cortinariaceae | 485/540(90%) | JQ724055 | Uncultured *Hebeloma* | -- |
| 145 | 1 | 8 | Basidiomycota | Thelephorales | Thelephoraceae | 452/453(99%) | JX630386 | Uncultured *Tomentella*^#^ | Root system in USA: AK (70.36 N 148.53 W) |
| 146 | 1 | 8 | Glomeromycota | Unassigned | Unassigned | 291/352(83%) | EF521235 | Uncultured fungus | -- |
|  |  |  |  |  |  | 136/140 (97%) | GU392007 | Uncultured Glomeromycota | Mycorrhizal associations of *Sarcochilus weinthalii* |
| 147 | 4 | 25 | Chytridiomycota | Chytridiales | Unassigned | 330/411(80%) | JX489834 | Uncultured soil fungus | -- |
|  |  |  |  |  |  | 163/173(94%) | EU352773 | Chytridiales sp. | -- |
| 148 | 1 | 3 | Unassigned | Unassigned | Unassigned | 200/257(78%) | FJ553066 | Uncultured *Venturia* | -- |
| 149 | 4 | 35 | Ascomycota | Capnodiales | Teratosphaeriaceae | 467/472(99%) | KF296772 | Uncultured fungus | Arctic soil in Canada (73.22 N 119.56 W) |
|  |  |  |  |  |  | 462/490(94%) | KF128864 | *Devriesia* sp. | -- |
| 150 | 4 | 37 | Ascomycota | Helotiales | Unassigned | 484/488(99%) | DQ182423 | Uncultured Ascomycota isolate | Plant roots |
|  |  |  |  |  |  | 488/497(98%) | KF646097 | *Leptodontidium orchidicola* ^#^ | Plant roots in Lithuania |
| 151 | 1 | 6 | Glomeromycota | Unassigned | Unassigned | 242/276(88%) | GU180306 | Uncultured fungus | -- |
|  |  |  |  |  |  | 163/185(88%) | JX898577 | Uncultured Glomeromycota | -- |
| 152 | 1 | 12 | Ascomycota | Hypocreales | Nectriaceae | 463/471(98%) | KC965805 | Uncultured fungus | Arctic soil in Canada (76.23 N 119.30 W) |
|  |  |  |  |  |  | 329/364(90%) | KT581658 | Uncultured *Cylindrium* | -- |
| 153 | 1 | 2 | Basidiomycota | Sporidiobolales | Unassigned | 430/433(99%) | KF297115 | Uncultured fungus | Arctic soil in Canada (76.23 N 119.30 W) |
|  |  |  |  |  |  | 379/435(87%) | NR073330 | *Rhodotorula ferulica* | -- |
| 154 | 2 | 28 | Basidiomycota | Thelephorales | Thelephoraceae | 474/476(99%) | KC966131 | Uncultured fungus | Arctic soil in USA (69.67 N 148.72 W) |
|  |  |  |  |  |  | 441/442(99%) | HQ215815 | *Tomentella* sp. ^#^ | Soil in Svalbard |
| 155 | 4 | 12 | Ascomycota | Helotiales | Unassigned | 380/410(93%) | KF274374 | Uncultured fungus | -- |
|  |  |  |  |  |  | 377/406(93%) | HQ845751 | Helotiales sp. | -- |
| 156 | 1 | 73 | Ascomycota | Pezizales | Pyronemataceae | 471/485(97%) | KF296914 | Uncultured fungus | Arctic soil in Canada (73.22 N 119.56 W) |
|  |  |  |  |  |  | 446/501(89%) | EU668294 | Uncultured Pyronemataceae | -- |
| 157 | 4 | 47 | Ascomycota | Verrucariales | Verrucariaceae | 350/364(96%) | KC966146 | Uncultured fungus | -- |
|  |  |  |  |  |  | 336/362(93%) | KF819520 | *Verrucaria devensis* | -- |
| 158 | 1 | 3 | Ascomycota | Orbiliales | Orbiliaceae | 429/429(100%) | KC966184 | Uncultured fungus | Arctic soil in USA (69.67 N 148.72 W) |
|  |  |  |  |  |  | 383/392(98%) | DQ494363 | *Dactylella* sp. ^#^ | Unreported |
| 159 | 4 | 10 | Ascomycota | Capnodiales | Cladosporiaceae | 492/494(99%) | LN808882 | *Cladosporium herbarum** | Air sample in Spain:Huelva, Gruta de las Maravillas |
| 160 | 2 | 18 | Glomeromycota | Glomerales | Unassigned | 302/355(85%) | KF297260 | Uncultured fungus | -- |
|  |  |  |  |  |  | 215/241(89%) | KF206536 | Uncultured Glomerales | -- |
| 161 | 3 | 86 | Ascomycota | Xylariales | Xylariaceae | 513/517(99%) | KT581873 | Uncultured Xylariaceae | Quercus deserticola litter in Mexico |
| 162 | 2 | 7 | Ascomycota | Lecanorales | Parmeliaceae | 393/395(99%) | DQ979999 | *Allantoparmelia alpicola** | In Sweden |
| 163 | 5 | 10 | Ascomycota | Hypocreales | Tilachlidiaceae | 490/495(99%) | KC966363 | Uncultured fungus | Arctic soil in Canada (73.22 N 119.56 W) |
|  |  |  |  |  |  | 446/484(92%) | KM231838 | *Tilachlidium brachiatum* | -- |
| 164 | 6 | 165 | Ascomycota | Helotiales | Unassigned | 460/513(90%) | DQ421214 | Uncultured soil fungus | -- |
|  |  |  |  |  |  | 458/515(89%) | EU883432 | *Tetracladium furcatum* | -- |
| 165 | 1 | 17 | Ascomycota | Helotiales | Hyaloscyphaceae | 496/531(93%) | HQ630988 | *Chalara* sp. | -- |
|  |  |  |  |  |  | 487/518(94%) | JF908571 | *Pezizella discreta* |  |
| 166 | 4 | 308 | Ascomycota | Helotiales | Unassigned | 481/527(91%) | KF742601 | Uncultured fungus | -- |
|  |  |  |  |  |  | 476/530(90%) | EU883433 | *Tetracladium* sp. | -- |
| 167 | 1 | 3 | Rozellomycota | Unassigned | Unassigned | 336/384(88%) | HM069429 | Uncultured fungus (Rozellomycota) | -- |
| 168 | 1 | 114 | Ascomycota | Helotiales | Unassigned | 485/542(89%) | KP889482 | Uncultured fungus | -- |
|  |  |  |  |  |  | 469/520(90%) | KM113762 | Helotiales sp. | -- |
| 169 | 5 | 525 | Ascomycota | Helotiales | Hyaloscyphaceae | 511/522(98%) | JF908571 | *Pezizella discreta*^#^ | Italy |
| 170 | 1 | 3 | Ascomycota | Verrucariales | Verrucariaceae | 452/466(97%) | EU249468 | *Thelidium methorium* | Switzerland |
| 171 | 2 | 41 | Basidiomycota | Thelephorales | Thelephoraceae | 465/474(98%) | JQ724032 | Uncultured Thelephoraceae | Ectomycorrhiza in Sweden (59.49 N 17.40 E) |
|  |  |  |  |  |  | 463/482(96%) | AJ534912 | *Tomentella* sp. ^#^ | -- |
| 172 | 1 | 2 | Ascomycota | Helotiales | Unassigned | 475/502(95%) | HF947846 | Uncultured Helotiales | -- |
| 173 | 1 | 37 | Basidiomycota | Thelephorales | Thelephoraceae | 467/478(98%) | JX630720 | Uncultured *Tomentella* | root system in USA: Happy Valley, AK (69.13 N 148.83 W) |
| 174 | 2 | 850 | Ascomycota | Capnodiales | Unassigned | 465/498(93%) | KM186816 | *Bryochiton perpusillus* | -- |
|  |  |  |  |  |  | 471/538(88%) | JF499843 | *Penidiella ellipsoiden* | -- |
| 175 | 1 | 9 | Ascomycota | Helotiales | Dermateaceae | 483/487(99%) | KF296725 | Uncultured fungus (Helotiales) | Arctic soil in Canada (76.23N, 119.30W) |
|  |  |  |  |  |  | 298/309(96%) | KM216323 | *Scleropezicula* sp. ^#^ | -- |
| 176 | 1 | 120 | Ascomycota | Helotiales | Unassigned | 509/535(95%) | KF742601 | Uncultured fungus | -- |
|  |  |  |  |  |  | 508/535(95%) | GU327472 | Uncultured *Tetracladium* ^#^ | -- |
| 177 | 7 | 263 | Ascomycota | Pleosporales | Diademaceae | 503/509(99%) | JX978255 | Fungal sp. | Plant twig tissue in USA (Utah) |
|  |  |  |  |  |  | 484/495(98%) | KP334718 | *Comoclathris sedi* ^#^ | Italy |
| 178 | 2 | 7 | Basidiomycota | Sebacinales | Sebacinaceae | 506/514(98%) | EU910924 | Uncultured *Sebacina* ^#^ | Mycobiont of *Trifolium pretense* |
| 179 | 4 | 147 | Ascomycota | Chaetothyriales | Herpotrichiellaceae | 538/553(97%) | KC965227 | Uncultured fungus | Arctic soil in Canada (76.23N, 119.30W) |
|  |  |  |  |  |  | 499/508(98%) | FJ948175 | *Rhinocladiella* sp. ^#^ | Rock in China |
| 180 | 2 | 25 | Ascomycota | Chaetothyriales | Herpotrichiellaceae | 508/529(96%) | KC965234 | Uncultured fungus | -- |
|  |  |  |  |  |  | 485/556(87%) | FJ475766 | Uncultured Herpotrichiellaceae | -- |
| 181 | 4 | 19 | Basidiomycota | Agaricales | Inocybaceae | 475/478(99%) | KF617469 | Uncultured fungus | Forest soil in USA (63.84N, 145.72W) |
|  |  |  |  |  |  | 458/461(99%) | HQ215793 | *Inocybe* sp. ^#^ | Soil |
| 182 | 7 | 73 | Basidiomycota | Cystofilobasidiales | Cystofilobasidiaceae | 469/470(99%) | AM901861 | Uncultured basidiomycete | House dust in Finland |
|  |  |  |  |  |  | 428/432(99%) | AY038826 | *Mrakia* sp. ^#^ | Antarctica |
| 183 | 2 | 10 | Ascomycota | Baeomycetales | Unassigned | 377/400(94%) | KC966183 | Uncultured fungus | -- |
|  |  |  |  |  |  | 382/432(88%) | KP698194 | *Sarea* sp. | -- |
| 184 | 5 | 13 | Ascomycota | Capnodiales | Unassigned | 507/514(99%) | KF297284 | Uncultured fungus | Arctic soil in Canada (73.22 N 119.56 W) |
|  |  |  |  |  |  | 480/543(88%) | AM746201 | *Capnobotryella* sp. | -- |
| 185 | 5 | 523 | Ascomycota | Verrucariales | Verrucariaceae | 511/515(99%) | KC965806 | Uncultured fungus | Arctic soil in Canada (76.23N, 119.30W) |
|  |  |  |  |  |  | 451/475(95%) | FJ232935 | *Atla* sp. ^#^ | -- |
| 186 | 2 | 20 | Zygomycota | Mortierellales | Unassigned | 398/404(99%) | KF617387 | Uncultured fungus | Forest soil in USA: Alaska (63.8466 N 145.7209 W) |
|  |  |  |  |  |  | 385/424(91%) | JQ272348 | Mortierellales sp. | -- |
| 187 | 9 | 608 | Ascomycota | Helotiales | Unassigned | 514/517(99%) | JQ666492 | Uncultured soil fungus | Forest soil in China: Changbai mountain |
|  |  |  |  |  |  | 464/479(97%) | FJ196296 | Helotiales sp. | Mycorrhiza in Taiwan |
| 188 | 2 | 17 | Ascomycota | Helotiales | Unassigned | 490/498(98%) | FJ553817 | Uncultured Helotiales | Forest soil in Canada |
|  |  |  |  |  |  | 470/486(97%) | DQ132821 | *Cadophora* sp. ^#^ | Rhizosphere of clonal *Picea mariana* plants |
| 189 | 1 | 3 | Ascomycota | Pleosporales | Unassigned | 414/419(99%) | KC965505 | Uncultured fungus | Arctic soil in USA (69.67 N 148.72 W) |
|  |  |  |  |  |  | 369/441(84%) | HQ631002 | Pleosporales sp. | -- |
| 190 | 2 | 13 | Ascomycota | Lecanorales | Unassigned | 428/458(93%) | KF297261 | Uncultured fungus (Lecanorales) | -- |
|  |  |  |  |  |  | 393/430(91%) | FJ008690 | Ascomycota sp. | -- |
| 191 | 1 | 98 | Unassigned | Unassigned | Unassigned | 217/242(90%) | KF297107 | Uncultured fungus | -- |
| 192 | 5 | 36 | Zygomycota | Mortierellales | Mortierellaceae | 471/473(99%) | KC965233 | Uncultured fungus | Arctic soil in Canada (76.23N, 119.30W) |
|  |  |  |  |  |  | 469/484(97%) | JX270427 | *Mortierella* sp. ^#^ | Soil in USA: New York |
| 193 | 9 | 973 | Ascomycota | Verrucariales | Verrucariaceae | 523/524(99%) | KC966360 | Uncultured fungus | Arctic soil in Canada (73.22 N 119.56 W) |
|  |  |  |  |  |  | 463/483(96%) | FJ232935 | *Atla* sp. ^#^ | -- |
| 194 | 4 | 24 | Ascomycota | Capnodiales | Unassigned | 373/394(95%) | KC965399 | Uncultured fungus | -- |
|  |  |  |  |  |  | 354/412(86%) | AJ971406 | *Capnobotryella* sp. | -- |
| 195 | 1 | 3 | Basidiomycota | Thelephorales | Thelephoraceae | 429/441(97%) | JX630720 | Uncultured *Tomentella* | Root system in USA: Happy Valley, AK (69.13 N 148.83 W) |
| 196 | 5 | 79 | Ascomycota | Chaetothyriales | Unassigned | 483/497(97%) | KC966034 | Uncultured fungus | Arctic soil in Canada (73.22 N 119.56 W) |
|  |  |  |  |  |  | 452/526(86%) | FJ553154 | Uncultured Herpotrichiellaceae | -- |
| 197 | 4 | 7 | Rozellomycota | Unassigned | Unassigned | 376/380(99%) | AF504874 | Uncultured fungus (Rozellomycota) | Soil |
| 198 | 1 | 2 | Zygomycota | Mortierellales | Mortierellaceae | 406/416(98%) | JX374816 | Uncultured fungus | Soil in USA |
|  |  |  |  |  |  | 385/396(97%) | AJ271629 | *Mortierella alpina*^#^ | Unreported |
| 199 | 1 | 16 | Basidiomycota | Thelephorales | Thelephoraceae | 479/489(98%) | JX907823 | Fungal sp. | Ectomycorrhizal root tip in Latvia |
|  |  |  |  |  |  | 469/489(96%) | JQ711817 | *Tomentella* sp. ^#^ | -- |
| 200 | 1 | 7 | Ascomycota | Capnodiales | Unassigned | 505/507(99%) | KF296999 | Uncultured fungus | Arctic soil in Canada (76.23N, 119.30W) |
|  |  |  |  |  |  | 360/414 (91%) | GQ852801 | *Teratosphaeria eucalypti* | -- |
| 201 | 3 | 53 | Ascomycota | Helotiales | Helotiaceae | 366/384(95%) | JQ666492 | Uncultured soil fungus | -- |
|  |  |  |  |  |  | 359/378(95%) | KC455359 | Uncultured Helotiaceae | -- |
| 202 | 1 | 5 | Chytridiomycota | Unassigned | Unassigned | 265/327(81%) | JX388918 | Uncultured fungus | -- |
|  |  |  |  |  |  | 129/137(94%) | DQ536497 | *Rhizophydium* sp. | -- |
| 203 | 1 | 5 | Ascomycota | Hypocreales | Unassigned | 348/368(95%) | KC965274 | Uncultured fungus | -- |
|  |  |  |  |  |  | 342/386(89%) | KM889547 | Uncultured *Nectria* | -- |
| 204 | 6 | 60 | Ascomycota | Pleosporales | Unassigned | 507/511(99%) | EU686520 | *Monodictys arctica** | Plant roots in the Canadian High Arctic |
|  |  |  |  |  |  | 508/514(99%) | JQ346922 | Uncultured *Ochrocladosporium* | Roots of herbs in alpine meadow (China) |
| 205 | 6 | 75 | Ascomycota | Helotiales | Unassigned | 496/496(100%) | KC965366 | Uncultured fungus | Arctic soil in Canada (76.23N, 119.30W) |
|  |  |  |  |  |  | 504/524(96%) | KR063520 | Uncultured *Tetracladium* ^#^ | -- |
| 206 | 1 | 10 | Ascomycota | Verrucariales | Verrucariaceae | 296/302(98%) | FJ664859 | *Verrucaria* sp. ^#^ | Iceland |
| 207 | 1 | 8 | Ascomycota | Helotiales | Vibrisseaceae | 432/443(98%) | FR774054 | Uncultured *Phialocephala* ^#^ | Leaf surface |
| 208 | 1 | 6 | Ascomycota | Helotiales | Unassigned | 379/395(96%) | GU174334 | Uncultured fungus | -- |
|  |  |  |  |  |  | 321/339(95%) | EU726288 | Uncultured Helotiales | -- |
| 209 | 2 | 60 | Ascomycota | Chaetothyriales | Herpotrichiellaceae | 533/543(98%) | KC965841 | Uncultured fungus | Arctic soil in USA (69.67 N 148.72 W) |
|  |  |  |  |  |  | 413/451 (91%) | FJ265748 | *Cladophialophora* sp. | -- |
| 210 | 2 | 76 | Ascomycota | Chaetothyriales | Unassigned | 526/529(99%) | KC966354 | Uncultured fungus | Arctic soil in Canada (76.23N, 119.30W) |
|  |  |  |  |  |  | 503/567(89%) | FJ554329 | Uncultured Herpotrichiellaceae | -- |
| 211 | 6 | 160 | Zygomycota | Mortierellales | Mortierellaceae | 504/507(99%) | GQ219843 | Uncultured Mortierellaceae | Soil in Germany: Thuringia, Hainich |
|  |  |  |  |  |  | 497/497(100%) | JX270406 | *Mortierella* sp. ^#^ | soil from bat hibernaculum inUSA: Massachusetts |
| 212 | 4 | 18 | Ascomycota | Verrucariales | Unassigned | 296/338(88%) | FJ479632 | *Placopyrenium formosum* | -- |
| 213 | 2 | 10 | Ascomycota | Lecanorales | Stereocaulaceae | 378/404(94%) | KC966034 | Uncultured fungus | -- |
|  |  |  |  |  |  | 286/299(95%) | DQ396893 | *Stereocaulon alpestre* ^#^ | -- |
| 214 | 1 | 256 | Ascomycota | Sordariales | Lasiosphaeriaceae | 501/505(99%) | KC965644 | Uncultured fungus | Arctic soil in Canada (76.23N, 119.30W) |
|  |  |  |  |  |  | 440/488(90%) | JN689974 | *Podospora* sp. | -- |
| 215 | 1 | 19 | Basidiomycota | Agaricales | Cortinariaceae | 323/330(98%) | JQ347076 | Uncultured *Cortinarius* ^#^ | roots of herbs (ectomycorrhiza) |
| 216 | 1 | 13 | Ascomycota | Unassigned | Unassigned | 502/508(99%) | KC965200 | Uncultured fungus | Arctic soil in USA( 69.67 N 148.72 W) |
|  |  |  |  |  |  | 424/532(80%) | KF959776 | *Verrucaria submersella* | -- |
| 217 | 1 | 11 | Ascomycota | Hypocreales | Nectriaceae | 400/402(99%) | HQ446010 | Uncultured fungus | In China |
|  |  |  |  |  |  | 328/365(90%) | GU931759 | Uncultured *Cylindrium* | House dust in Canada (45.25N, 75.92W) |
| 218 | 10 | 1091 | Ascomycota | Helotiales | Unassigned | 498/508(98%) | KC965311 | Uncultured fungus | Arctic soil in Canada (73.22 N 119.56 W) |
|  |  |  |  |  |  | 490/521(94%) | KC694156 | Uncultured *Tetracladium* | -- |
| 219 | 5 | 1115 | Glomeromycota | Unassigned | Unassigned | 477/482(99%) | KC966163 | Uncultured fungus | Arctic soil in Canada (78.78 N 103.55 W) |
|  |  |  |  |  |  | 484/513(94%) | GU998151 | Uncultured glomeromycete | -- |
| 220 | 1 | 138 | Ascomycota | Arthoniales | Arthoniaceae | 467/498(94%) | KC965222 | Uncultured fungus | -- |
|  |  |  |  |  |  | 281/318(88%) | FR799123 | *Arthonia patellulata* | -- |
| 221 | 3 | 59 | Ascomycota | Pezizales | Unassigned | 471/473(99%) | KF617909 | Uncultured fungus | Forest soil in USA: Alaska (64.8659 N 147.8737 W) |
|  |  |  |  |  |  | 189/199(95%) | FJ553691 | Uncultured Pyronemataceae | -- |
| 222 | 2 | 21 | Basidiomycota | Sebacinales | Sebacinaceae | 374/375(99%) | JX630403 | Uncultured *Sebacina* ^#^ | Root system in USA: Westdock, AK (70.36 N 148.53 W) |
| 223 | 3 | 15 | Ascomycota | Helotiales | Unassigned | 491/493(99%) | KC965927 | Uncultured fungus | Arctic soil in Canada (76.23N, 119.30W) |
|  |  |  |  |  |  | 448/478 (94%) | FJ196296 | Helotiales sp. | Mycorrhiza in Taiwan |
| 224 | 3 | 58 | Ascomycota | Lecideales | Unassigned | 519/523(99%) | KF297187 | Uncultured fungus | Arctic soil in Canada (76.23N, 119.30W) |
|  |  |  |  |  |  | 182/185(98%) | AM292701 | *Lecidea sphaerella* ^#^ | Unreported |
| 225 | 1 | 68 | Basidiomycota | Tremellales | Unassigned | 495/517(96%) | GU328576 | Uncultured Basidiomycota | -- |
|  |  |  |  |  |  | 461/478(96%) | NR073211 | *Cryptococcus skinneri* ^#^ | -- |
| 226 | 4 | 445 | Ascomycota | Capnodiales | Unassigned | 377/444(85%) | KC965399 | Uncultured fungus | -- |
|  |  |  |  |  |  | 311/366(85%) | HM012872 | *Mycosphaerella swartii* | -- |
| 227 | 2 | 4 | Ascomycota | Helotiales | Unassigned | 395/396(99%) | KC965959 | Uncultured fungus | Arctic soil in Canada (73.22 N 119.56 W) |
|  |  |  |  |  |  | 379/421(90%) | FJ553913 | Uncultured *Tetracladium* | -- |
| 228 | 1 | 6 | Ascomycota | Unassigned | Unassigned | 383/387(99%) | KC965506 | Uncultured fungus | Arctic soil in USA ( 69.67 N 148.72 W) |
|  |  |  |  |  |  | 272/331(82%) | FJ479636 | *Placopyrenium fuscellum* | -- |
| 229 | 1 | 28 | Basidiomycota | Agaricales | Unassigned | 382/443(86%) | EU489977 | Uncultured Basidiomycota | -- |
|  |  |  |  |  |  | 375/436(86%) | FJ475673 | Uncultured Agaricales clone | -- |
| 230 | 1 | 15 | Ascomycota | Verrucariales | Verrucariaceae | 497/504(99%) | KC965812 | Uncultured fungus | Arctic soil in Canada (73.22 N 119.56 W) |
|  |  |  |  |  |  | 477/478 (99%) | EU559741 | *Polyblastia sendtneri** | Sweden |
| 231 | 1 | 5 | Chytridiomycota | Rhizophydiales | Unassigned | 241/280(86%) | EU517004 | Uncultured fungus | -- |
|  |  |  |  |  |  | 178/190(94%) | DQ485630 | *Rhizophydium* sp. | -- |
| 232 | 5 | 18 | Ascomycota | Helotiales | Unassigned | 526/528(99%) | HM116747 | *Cadophora luteo-olivacea** | Plant (*Vitis vinifera*) n New Zealand |
| 233 | 2 | 9 | Ascomycota | Unassigned | Unassigned | 274/337(81%) | KF937349 | *Ophiocordyceps australis* | -- |
| 234 | 3 | 22 | Ascomycota | Helotiales | Hyaloscyphaceae | 402/421(95%) | JX371639 | Uncultured fungus | -- |
|  |  |  |  |  |  | 393/410(96%) | JF908571 | *Pezizella discrete* ^#^ | -- |
| 235 | 2 | 21 | Basidiomycota | Filobasidiales | Unassigned | 478/478(100%) | KM504413 | Uncultured fungus | Ectomycorrhizae in Austria: Haggen |
|  |  |  |  |  |  | 478/478 (100%) | HE863717 | *Cryptococcus terricola** | Soil in Svalbard: Longyearbyen (78.24N, 15.49E) |
| 236 | 5 | 50 | Basidiomycota | Tremellales | Unassigned | 482/484(99%) | KC753404 | Uncultured *Cryptococcus*^#^ | Grain in Sweden |
|  |  |  |  |  |  | 464/468(99%) | AJ581048 | *Cryptococcus victoriae** | Plant roots in Germany |
| 237 | 1 | 68 | Ascomycota | Acarosporales | Unassigned | 486/533(91%) | HM069490 | Uncultured fungus (Acarosporales) | -- |
| 238 | 1 | 12 | Unassigned | Unassigned | Unassigned | 490/497(99%) | JQ247401 | Uncultured Dikarya | Arid and semiarid soils of Baja California, Mexico |
|  |  |  |  |  |  | 209/218(96%) | AY749465 | Uncultured fungus | -- |
| 239 | 1 | 2 | Ascomycota | Unassigned | Unassigned | 344/392(88%) | KC966239 | Uncultured fungus | -- |
|  |  |  |  |  |  | 317/378(84%) | KM062108 | Uncultured *Neoscytalidium* | -- |
| 240 | 4 | 693 | Basidiomycota | Agaricales | Cortinariaceae | 483/489(99%) | EU525959 | *Cortinarius cf. multiformis** | Ectomycorrhiza in USA: Oregon |
| 241 | 4 | 19 | Ascomycota | Helotiales | Unassigned | 513/519(99%) | HG327912 | Uncultured fungus | Arable soil in China:Gongzhuling |
|  |  |  |  |  |  | 511/520(98%) | JN859275 | Helotiales sp. | Plant root in Hungary (47.06 N 19.40 E) |
| 242 | 1 | 17 | Chytridiomycota | Unassigned | Unassigned | 320/370(86%) | JX388918 | Uncultured fungus | -- |
|  |  |  |  |  |  | 312/366(85%) | HQ191313 | Uncultured Chytridiomycota | -- |
| 243 | 1 | 6 | Basidiomycota | Sebacinales | Sebacinaceae | 405/408(99%) | GU327499 | Uncultured Sebacinales | Mycorrhizal seedling in Czech Republic |
|  |  |  |  |  |  | 402/410(98%) | KF000673 | Uncultured *Sebacina*^#^ | Ectomycorrhizal root tip in Germany |
| 244 | 4 | 79 | Ascomycota | Helotiales | Unassigned | 482/484(99%) | JX029114 | *Tetracladium* sp. | Qinghai-Tibet plateau |
|  |  |  |  |  |  | 483/500(97%) | FJ554342 | Uncultured Ascomycota | Forest soil in Canada |
| 245 | 8 | 134 | Ascomycota | Helotiales | Helotiaceae | 377/386(98%) | JQ666492 | Uncultured soil fungus | Forest soil in China: Changbai mountain |
|  |  |  |  |  |  | 381/394(97%) | KC455359 | Uncultured Helotiaceae | Root system in USA :AK |
| 246 | 1 | 6 | Ascomycota | Minutisphaerales | Minutisphaeraceae | 464/481(96%) | KF617742 | Uncultured fungus | -- |
|  |  |  |  |  |  | 434/454(95%) | AB733435 | *Minutisphaera japonica* ^#^ | -- |
| 247 | 1 | 142 | Basidiomycota | Agaricales | Cortinariaceae | 480/486(99%) | AM902090 | Uncultured basidiomycete | house dust in Finland |
|  |  |  |  |  |  | 480/487(99%) | JQ711769 | *Cortinarius* sp. ^#^ | Ectomycorrhiza in Canada: BC (55.45 N 123.20 W) |
| 248 | 2 | 140 | Unassigned | Unassigned | Unassigned | 191/209(91%) | KF297107 | Uncultured fungus | -- |
| 249 | 3 | 556 | Ascomycota | Helotiales | Unassigned | 507/515(98%) | KF617962 | Uncultured fungus (Helotiales) | Forest soil in USA: Alaska (63.90 N 145.37 W) |
|  |  |  |  |  |  | 519/537(97%) | FJ475721 | Uncultured Ascomycota (Helotiales) | *Pinus sylvestris* forest soil in Sweden |
| 250 | 5 | 1289 | Ascomycota | Chaetothyriales | Unassigned | 469/470(99%) | KC966034 | Uncultured fungus | Arctic soil in Canada (76.23N, 119.30W) |
|  |  |  |  |  |  | 437/507(86%) | FJ553154 | Uncultured Herpotrichiellaceae | -- |
| 251 | 1 | 22 | Basidiomycota | Unassigned | Unassigned | 417/489(85%) | EU489977 | Uncultured Basidiomycota | -- |
| 252 | 7 | 113 | Ascomycota | Hypocreales | Nectriaceae | 593/601(99%) | FN397215 | Uncultured fungus | Soil,burnt area in France:Cahors (44.26 N 1.26 E) |
|  |  |  |  |  |  | 588/602(98%) | JX915249 | *Fusarium verticillioides* ^#^ | Soil |
| 253 | 8 | 1064 | Ascomycota | Helotiales | Unassigned | 474/479(99%) | JX338645 | Uncultured fungus | Soil |
|  |  |  |  |  |  | 475/485(98%) | KR063520 | Uncultured *Tetracladium* ^#^ | Root China: Gansu, Lanzhou |
| 254 | 2 | 3 | Ascomycota | Dothideales | Aureobasidiaceae | 594/600(99%) | JX984782 | Uncultured fungus | TSP in urban air in South Korea: Seoul |
|  |  |  |  |  |  | 588/600(98%) | JF439462 | *Aureobasidium pullulans*^#^ | Soil in China (Zijin Mountain) |
| 255 | 1 | 5 | Glomeromycota | Unassigned | Unassigned | 446/467(96%) | EF521235 | Uncultured fungus | -- |
|  |  |  |  |  |  | 376/395(95%) | EF619903 | Uncultured Glomeromycota | -- |
| 256 | 7 | 111 | Ascomycota | Helotiales | Helotiaceae | 492/499(99%) | KC965406 | Uncultured fungus | Arctic soil in Canada (76.23N, 119.30W) |
|  |  |  |  |  |  | 477/496 (96%) | AY204609 | *Tricladium angulatum*^#^ | -- |
| 257 | 2 | 15 | Ascomycota | Verrucariales | Verrucariaceae | 353/369(96%) | KC966180 | Uncultured fungus | -- |
|  |  |  |  |  |  | 309/340(91%) | JQ088050 | *Polyblastia sendtneri* | -- |
| 258 | 2 | 4 | Ascomycota | Pezizales | Pyronemataceae | 482/500(96%) | KF617920 | Uncultured fungus | -- |
|  |  |  |  |  |  | 469/519(90%) | GU222313 | *Aleuria* sp. | -- |
| 259 | 1 | 4 | Basidiomycota | Tremellales | Tremellaceae | 448/450(99%) | KM504421 | Uncultured fungus | Ectomycorrhizae in subalpine soil in Austria: Haggen |
|  |  |  |  |  |  | 410/412(99%) | JX092261 | *Cryptococcus friedmannii** | Superficial sediments Italy: Sforzellina Glacier, Italian Alps |
| 260 | 2 | 19 | Chytridiomycota | Unassigned | Unassigned | 372/396(94%) | KF296968 | Uncultured fungus | -- |
|  |  |  |  |  |  | 292/358(82%) | HQ191313 | Uncultured Chytridiomycota | -- |
| 261 | 3 | 13 | Basidiomycota | Malasseziales | Malasseziaceae | 445/446(99%) | DQ279840 | Uncultured fungus | Deep sea sediment in China: South China Sea |
|  |  |  |  |  |  | 444/446(99%) | EU400587 | *Malassezia restricta** | Puccinia horiana pustule |
| 262 | 1 | 2 | Ascomycota | Capnodiales | Teratosphaeriaceae | 405/444(91%) | KC965399 | Uncultured fungus | -- |
|  |  |  |  |  |  | 317/370(86%) | GQ890348 | *Teratosphaeria crispata* | Eucalypts in eastern Australia |
| 263 | 1 | 7 | Basidiomycota | Unassigned | unassigned | 444/492(90%) | KC965726 | Uncultured fungus | -- |
|  |  |  |  |  |  | 458/527(87%) | KF225852 | Uncultured *Cryptococcus* | -- |
| 264 | 2 | 16 | Ascomycota | Pleosporales | Unassigned | 380/387(98%) | KC966185 | Uncultured fungus (Pleosporales) | Arctic soil in USA ( 69.67 N 148.72 W) |
|  |  |  |  |  |  | 345/428(81%) | HQ631002 | Pleosporales sp. | -- |
| 265 | 10 | 128 | Ascomycota | Helotiales | Unassigned | 517/527(98%) | AM901833 | Uncultured ascomycete | House dust in Finland |
|  |  |  |  |  |  | 512/527(97%) | EU883432 | *Tetracladium furcatum*^#^ | Aquatic hyphomycetes |
| 266 | 4 | 22 | Ascomycota | Pezizales | Pyronemataceae | 461/481(96%) | JX630433 | Uncultured *Geopora* ^#^ | -- |
| 267 | 1 | 5 | Ascomycota | Pleosporales | Unassigend | 320/327(98%) | EU516991 | Uncultured fungus (Pleosporales) | Soil in Austria |
|  |  |  |  |  |  | 263/281(94%) | DQ182451 | Uncultured Pleosporales | -- |
| 268 | 2 | 16 | Ascomycota | Helotiaceae | Helotiaceae | 384/422(91%) | FJ440900 | Uncultured Helotiaceae | -- |
| 269 | 3 | 31 | Ascomycota | Unassigned | Unassigned | 367/447(82%) | HQ631006 | Pleosporales sp. | -- |
| 270 | 4 | 38 | Ascomycota | Unassigned | Unassigned | 291/362(80%) | AB598100 | Helotiales sp. | -- |
| 271 | 1 | 3 | Glomeromycota | Unassigned | Unassigned | 328/406(81%) | HQ257445 | Uncultured fungus | -- |
|  |  |  |  |  |  | 197/221(89%) | EF619903 | Uncultured Glomeromycota | -- |
| 272 | 2 | 26 | Rozellomycota | Unassigned | Unassigned | 237/256(92%) | KC965536 | Uncultured fungus (Rozellomycota) | -- |
| 273 | 8 | 53 | Ascomycota | Eurotiales | Trichocomaceae | 538/540(99%) | HM801881 | *Aspergillus* sp. ^#^ | Rock phosphate mine landfills |
| 274 | 3 | 24 | Ascomycota | Helotiales | Unassigned | 503/506(99%) | JQ666493 | Uncultured soil fungus | Forest soil in China: Changbai mountain |
|  |  |  |  |  |  | 446/450(99%) | KC485427 | Helotiales sp. | Algae in Antarctica |
| 275 | 1 | 306 | Ascomycota | Sordariales | Unassigned | 482/504(96%) | FJ554320 | Uncultured Sordariomycetes | -- |
|  |  |  |  |  |  | 472/493(96%) | JN704818. | Uncultured Sordariales | -- |
| 276 | 1 | 4 | Ascomycota | Helotiales | Unassigned | 492/503(98%) | KF274367 | Uncultured fungus | Wood stump in Finland |
|  |  |  |  |  |  | 474/489(97%) | HQ608110 | Helotiales sp. | Nest in USA: Stengl Biology Station, Texas |
| 277 | 1 | 79 | Ascomycota | Helotiales | Unassigned | 483/485(99%) | HQ445248 | Uncultured fungus | Root of alpine and arctic Dryas octopetala |
|  |  |  |  |  |  | 350/369(95%) | KF730849 | *Varicosporium delicatum*^#^ | -- |
| 278 | 2 | 65 | Chytridiomycota | Unassigned | Unassigned | 507/513(99%) | KF296736 | Uncultured fungus | Arctic soil in Canada (76.23N, 119.30W) |
|  |  |  |  |  |  | 216/226 (95%) | EU490042 | Uncultured Chytridiomycota | -- |
| 279 | 1 | 3 | Chytridiomycota | Unassigned | Unassigned | 408/413(99%) | KF296886 | Uncultured fungus | Arctic soil in Canada (73.22N, 119.56W) |
|  |  |  |  |  |  | 337/397(85%) | HQ219421 | Uncultured Chytridiomycota | Unreported |
| 280 | 4 | 11 | Ascomycota | Chaetothyriales | Herpotrichiellaceae | 412/421(98%) | KC965460 | Uncultured fungus | Arctic soil in Canada (76.23N, 119.30W) |
|  |  |  |  |  |  | 370/389(95%) | FJ948175 | *Rhinocladiella* sp. ^#^ | -- |
| 281 | 8 | 841 | Ascomycota | Helotiales | Unassigned | 524/534(98%) | KC694156 | Uncultured *Tetracladium* ^#^ | Plant roots in Sweden |
| 282 | 2 | 131 | Ascomycota | Sordariales | Lasiosphaeriaceae | 471/494(95%) | FJ552801 | Uncultured Sordariomycetes | -- |
|  |  |  |  |  |  | 464/498(93%) | HQ631039 | *Cercophora* sp. | -- |
| 283 | 1 | 2279 | Basidiomycota | Agaricales | Tricholomataceae | 507/511(99%) | JQ666645 | Uncultured soil fungus | Forest soil in China (Changbai mountain) |
|  |  |  |  |  |  | 483/493(98%) | JF908413 | *Mycena maurella* ^#^ | Italy |
| 284 | 1 | 34 | Ascomycota | Helotiales | Unassigned | 443/482(92%) | KF296998 | Uncultured fungus | -- |
|  |  |  |  |  |  | 429/482(89%) | KM113762 | Helotiales sp. | -- |
| 285 | 2 | 9 | Chytridiomycota | Chytridiales | Unassigned | 304/364(84%) | JX344420 | Uncultured fungus | -- |
|  |  |  |  |  |  | 235/248 (94%) | EF432821 | Chytridiales sp. | -- |
| 286 | 2 | 19 | Ascomycota | Peltigerales | Collemataceae | 349/420(83%) | KC965657 | Uncultured fungus | -- |
|  |  |  |  |  |  | 212/227(93%) | DQ466035 | *Leptogium quercicola* | -- |
| 287 | 1 | 8 | Chytridiomycota | Rhizophydiales | Unassigned | 373/448(83%) | KC965876 | Uncultured fungus | -- |
|  |  |  |  |  |  | 244/266(91%) | DQ485617 | *Rhizophydium* sp. | -- |
| 288 | 1 | 8 | Basidiomycota | Agaricales | Inocybaceae | 276/290(95%) | FJ904129 | *Inocybe cf. flavella* ^#^ | -- |
| 289 | 1 | 49 | Ascomycota | Pezizales | Unassigned | 447/509(88%) | AF064596 | *Wynnella silvicola* | -- |
| 290 | 3 | 10 | Ascomycota | Coniochaetales | Coniochaetaceae | 452/487(93%) | KF823594 | Uncultured *Coniochaeta* | -- |
|  |  |  |  |  |  | 437/476(92%) | GU062217 | *Lecythophora* sp. | -- |
| 291 | 6 | 152 | Ascomycota | Dothideales | Unassigned | 521/525(99%) | KF527818 | Fungal sp. | Arctic plant roots |
|  |  |  |  |  |  | 499/508(98%) | KJ735004 | *Neopeckia* sp. ^#^ | Plateau grassland soil in Tibet |
| 292 | 7 | 53 | Ascomycota | Hypocreales | Nectriaceae | 531/533(99%) | JF735314 | *Neonectria ramulariae** | Fruit (*Malus sylvestris*) |
| 293 | 2 | 16 | Basidiomycota | Agaricales | Inocybaceae | 329/333(99%) | HQ215782 | *Inocybe* sp. | Soil in Svalbard |
| 294 | 5 | 11 | Ascomycota | Pleosporales | Pleosporaceae | 543/544(99%) | KP739874 | *Alternaria alternata** | Chukchi Sea grounds |
| 295 | 3 | 1044 | Basidiomycota | Sebacinales | Sebacinaceae | 484/495(98%) | GQ219874 | Uncultured *Sebacina* ^#^ | Soil in Gemany |
| 296 | 6 | 127 | Ascomycota | Chaetothyriales | Unassigned | 392/403(97%) | KC966034 | Uncultured fungus | Arctic soil in Canada (73.22N, 119.56W) |
|  |  |  |  |  |  | 389/403(97%) | KC965317 | Uncultured fungus (Chaetothyriales) | -- |
| 297 | 1 | 6 | Basidiomycota | Thelephorales | Thelephoraceae | 428/431(99%) | KF617907 | Uncultured fungus | Forest soil in Alaska (64.86N, 147.87W) |
|  |  |  |  |  |  | 389/390 (99%) | HQ215814 | *Tomentella* sp. ^#^ | Soil in Svalbard |
| 298 | 1 | 14 | Ascomycota | Helotiales | Unassigend | 482/507(95%) | HG327912 | Uncultured fungus | -- |
|  |  |  |  |  |  | 455/479 (94%) | JN859275 | Helotiales sp. | -- |
| 299 | 1 | 19 | Ascomycota | Umbilicariales | Unassigned | 407/415(98%) | KF296851 | Uncultured fungus | Arctic soil in Canada (73.22N, 119.56W) |
|  |  |  |  |  |  | 374/442(85%) | AF096208 | *Umbilicaria vellea* | -- |
| 300 | 1 | 6 | Rozellomycota | Unassigned | Unassigned | 468/475(99%) | KP889857 | Uncultured fungus | Forest soil in Canada |
|  |  |  |  |  |  | 358/393(91%) | EF635746 | Uncultured fungus (Rozellomycota) | -- |
| 301 | 1 | 10 | Unassigned | Unassigned | Unassigned | 340/419(81%) | KF297253 | Uncultured fungus | -- |
| 302 | 6 | 45 | Ascomycota | Helotiales | Helotiaceae | 387/393(98%) | KC965406 | Uncultured fungus | Arctic soil in Canada (76.23N, 119.30W) |
|  |  |  |  |  |  | 367/389(94%) | AY204609 | *Tricladium angulatum* | -- |
| 303 | 5 | 101 | Ascomycota | Umbilicariales | Unassigned | 391/417(94%) | KF296938 | Uncultured fungus | -- |
|  |  |  |  |  |  | 397/461(86%) | AF096211 | *Umbilicaria leiocarpa* | -- |
| 304 | 3 | 111 | Basidiomycota | Sebacinales | Sebacinaceae | 513/514(99%) | FJ553298 | Uncultured Sebacinales | Forest soil in Canada |
|  |  |  |  |  |  | 481/490(98%) | JX844772 | Uncultured *Sebacina* ^#^ | Mycorrhizal root zip |
| 305 | 1 | 2 | Ascomycota | Unassigned | Unassigned | 333/356(94%) | FJ197894 | Uncultured fungus | -- |
|  |  |  |  |  |  | 350/438(80%) | AJ879654 | Uncultured Dermataceae | -- |
| 306 | 1 | 5 | Ascomycota | Chaetothyriales | Herpotrichiellaceae | 391/424(92%) | FJ552708 | Uncultured Herpotrichiellaceae | -- |
| 307 | 4 | 436 | Ascomycota | Helotiales | Hyaloscyphaceae | 402/419(96%) | JX321070 | Uncultured fungus | -- |
|  |  |  |  |  |  | 392/409(96%) | JF908571 | *Pezizella discrete* ^#^ | -- |
| 308 | 3 | 220 | Chytridiomycota | Unassigned | Unassigned | 293/356(82%) | GU065483 | Uncultured fungus | -- |
|  |  |  |  |  |  | 280/354(79%) | HQ191313 | Uncultued Chytridiomycota | -- |
| 309 | 1 | 5 | Unassigned | Unassigned | Unassigned | 432/470(92%) | KF297047 | Uncultured fungus | -- |
| 310 | 1 | 4 | Chytridiomycota | Unassigned | Unassigned | 371/437(85%) | KF296968 | Uncultured fungus | -- |
|  |  |  |  |  |  | 269/242(94%) | DQ536497 | Uncultured Chytridiomycota | -- |
| 311 | 1 | 24 | Basidiomycota | Tremellales | Unassigned | 483/516(94%) | GU328576 | Uncultured Basidiomycota | -- |
|  |  |  |  |  |  | 423/442(96%) | JX268525 | *Cryptococcus* sp. ^#^ | -- |
| 312 | 5 | 270 | Ascomycota | Unassigned | Unassigned | 529/535(99%) | KM877207 | Uncultured fungus | Rain water in Tibet |
|  |  |  |  |  |  | 527/535(99%) | JF439476 | *Geomyces* sp. ^#^ | Zijing mountain, China |
| 313 | 3 | 18 | Ascomycota | Hypocreales | Nectriaceae | 533/533(100%) | KF576625 | *Fusarium cortaderiae** | Grain in Spain |
| 314 | 1 | 86 | Ascomycota | Unassigned | Unassigned | 460/483(95%) | KC965747 | Uncultured fungus | -- |
|  |  |  |  |  |  | 358/428 (84%) | GU067746 | Ascomycota sp. | -- |
| 315 | 1 | 8 | Basidiomycota | Cantharellales | Tulasnellaceae | 328/330(99%) | KC455339 | Uncultured Cantharellales | Plant roots in Arctic tundra (Alaska) |
|  |  |  |  |  |  | 287/298(96%) | DQ925585 | Uncultured Tulasnellaceae | -- |
| 316 | 1 | 35 | Basidiomycota | Agaricales | Tricholomataceae | 268/273(98%) | JQ666645 | Uncultured soil fungus | Forest soil in China (Changbai mountain) |
|  |  |  |  |  |  | 241/250(96%) | JF908395 | *Mycena speirea*^#^ | -- |
| 317 | 1 | 29 | Ascomycota | Helotiales | Unassigned | 462/507(91%) | HQ845751 | Helotiales sp. | -- |
| 318 | 6 | 84 | Basidiomycota | Kriegeriales | Kriegeriaceae | 507/512(99%) | KF274089 | Uncultured fungus | Wood stump in Finland |
|  |  |  |  |  |  | 472/479(96%) | EF151249 | *Rhodotorula glacialis*^#^ | -- |
| 319 | 2 | 39 | Glomeromycota | Unassigned | Unassigned | 205/230(89%) | JX372940 | Uncultured fungus | -- |
|  |  |  |  |  |  | 183/216(85%) | EF619905 | Uncultured Glomeromycota | -- |
| 320 | 2 | 381 | Basidiomycota | Agaricales | Unassigned | 462/525(88%) | EU489977 | Uncultured Basidiomycota | -- |
|  |  |  |  |  |  | 437/493(89%) | GU234093 | *Omphalina rustica* | -- |
| 321 | 2 | 3 | Ascomycota | Pleosporales | Phaeosphaeriaceae | 463/477(97%) | AM901822 | Uncultured ascomycete | House dust in Finland |
|  |  |  |  |  |  | 406/417(97%) | AF439507 | *Phaeosphaeria triglochinicola* ^#^ | *Triglochin palustris* in Switzerland |
| 322 | 1 | 2 | Ascomycota | Helotiales | Unassigned | 406/417(97%) | JX381513 | Uncultured fungus | Soil in USA |
|  |  |  |  |  |  | 449/480(94%) | JF748081 | Uncultured Helotiales | -- |
| 323 | 5 | 47 | Ascomycota | Helotiales | Unassigned | 360/365(99%) | KC965311 | Uncultured fungus | Arctic soil in Canada (73.22N, 119.56W) |
|  |  |  |  |  |  | 365/387(94%) | GU327472 | Uncultured *Tetracladium* |  |
| 324 | 1 | 4 | Zygomycota | Mortierellales | Mortierellaceae | 354/355(99%) | KF297122 | Uncultured fungus | Arctic soil in Canada (76.23N, 119.30W) |
|  |  |  |  |  |  | 352/369(95%) | JX270478 | *Mortierella* sp. ^#^ | -- |
| 325 | 3 | 27 | Ascomycota | Peltigerales | Collemataceae | 252/273(92%) | DQ466035 | *Leptogium quercicola* | -- |
| 326 | 2 | 5 | Ascomycota | Pleosporales | Torulaceae | 427/451(95%) | AY929129 | Uncultured mycorrhizal fungus | -- |
|  |  |  |  |  |  | 385/415(93%) | JN578618 | *Dendryphion penicillatum* | -- |
| 327 | 1 | 2 | Basidiomycota | Sebacinales | Unassigned | 304/327(93%) | GU083291 | Uncultured soil fungus | -- |
|  |  |  |  |  |  | 322/368(88%) | FJ788809 | Uncultured Sebacinales | -- |
| 328 | 1 | 77 | Unassigned | Unassigned | Unassigned | 223/238(94%) | EU003080 | Uncultured eukaryote | -- |
| 329 | 2 | 12 | Ascomycota | Chaetothyriales | Unassigned | 486/532(91%) | KC965841 | Uncultured fungus | -- |
|  |  |  |  |  |  | 450/505(89%) | EU139151 | *Capronia* sp. | -- |
| 330 | 1 | 11 | Chytridiomycota | Unassigned | Unassigned | 250/309(81%) | JF439198 | Fungal sp. | -- |
|  |  |  |  |  |  | 192/201(95%) | HQ191361 | Uncultured Chytridiomycota | -- |
| 331 | 4 | 63 | Ascomycota | Capnodiales | Unassigned | 397/422(94%) | JQ247386 | Uncultured fungus | -- |
|  |  |  |  |  |  | 377/403(94%) | JF499843 | *Penidiella ellipsoidea* | -- |
| 332 | 2 | 5 | Ascomycota | Chaetothyriales | Herpotrichiellaceae | 343/353(97%) | JF737788 | Uncultured fungus | Rock in China |
|  |  |  |  |  |  | 337/351(96%) | JX839531 | *Rhinocladiella* sp. ^#^ | -- |
| 333 | 7 | 113 | Ascomycota | Verrucariales | Verrucariaceae | 492/506(97%) | KC966180 | Uncultured fungus | Arctic soil in USA (69.67N, 148.72W) |
|  |  |  |  |  |  | 437/481(91%) | EU559739 | *Polyblastia* sp. | -- |
| 334 | 1 | 21 | Ascomycota | Hypocreales | Unassigned | 433/466(93%) | GQ924068 | Uncultured fungus | -- |
|  |  |  |  |  |  | 377/421(90%) | HQ389463 | Uncultured Hypocreales | -- |
| 335 | 1 | 7 | Basidiomycota | Tremellales | Unassigned | 341/370(92%) | FJ553848 | Uncultured Tremellales | -- |
| 336 | 5 | 20 | Ascomycotaa | Venturiales | Venturiaceae | 496/503(99%) | FJ553146 | Uncultured *Venturia* ^#^ | Forest soil in Canada |
| 337 | 3 | 9 | Ascomycota | Chaetothyriales | Unassigned | 208/213(98%) | KC965460 | Uncultured fungus | Arctic soil n Canada (76.23 N 119.30 W) |
|  |  |  |  |  |  | 158/164(96%) | FJ948175 | *Rhinocladiella* sp. ^#^ | -- |
| 338 | 1 | 17 | Unassigned | Unassigned | Unassigned | 434/460(94%) | EU516784 | Uncultured fungus | -- |
| 339 | 2 | 6 | Ascomycota | Helotiales | Hyaloscyphaceae | 528/529(99%) | FJ553766 | Uncultured Helotiales | Forest soil in Canada |
|  |  |  |  |  |  | 513/533(96%) | GU393951 | Hyaloscyphaceae sp. | -- |
| 340 | 1 | 12 | Ascomycota | Helotiales | Unassigned | 463/465(99%) | FJ378851 | Uncultured Helotiales | Ectomycorrhiza in eastern Himalaya |
| 341 | 3 | 10 | Ascomycota | Eurotiales | Aspergillaceae | 247/247(100%) | KP784374 | *Aspergillus flavus** | Coffee beans |
| 342 | 5 | 60 | Basidiomycota | Sebacinales | Sebacinaceae | 450/485(93%) | KC986274 | Uncultured *Sebacina* | -- |
| 343 | 1 | 24 | Chytridiomycota | Unassigned | Unassigned | 394/420(94%) | KF296968 | Uncultured fungus | -- |
|  |  |  |  |  |  | 299/364(82%) | HQ191313 | Uncultured Chytridiomycota | -- |
| 344 | 1 | 31 | Ascomycota | Saccharomycetales | Unassigned | 480/512(94%) | KF297139 | Uncultured fungus | -- |
|  |  |  |  |  |  | 185/201(92%) | JN183452 | *Candida bituminiphila* | -- |
| 345 | 1 | 14 | Glomeromycota | Unassigned | Unassigned | 262/270(97%) | JF300413 | Uncultured fungus | boreal forest soil in Sweden |
|  |  |  |  |  |  | 231/251(92%) | GU392007 | Uncultured Glomeromycota | -- |
| 346 | 2 | 340 | Ascomycota | Sordariales | Unassigned | 448/536(84%) | HM069420 | Uncultured fungus | -- |
|  |  |  |  |  |  | 364/421 (86%) | AB278194 | *Cephalotheca sulfurea* | -- |
| 347 | 4 | 99 | Basidiomycota | Agaricales | Cortinariaceae | 481/486(99%) | JX135072 | Uncultured *Hebeloma* ^#^ | Poplar roots |
| 348 | 7 | 108 | Ascomycota | Helotiales | Unassigned | 503/517(97%) | KC966026 | Uncultured fungus | Arctic soil in Canada (76.23N, 119.30W) |
|  |  |  |  |  |  | 476/494 (96%) | GU327472 | Uncultured *Tetracladium* ^#^ | -- |
| 349 | 1 | 194 | Basidiomycota | Thelephorales | Thelephoraceae | 477/480(99%) | FM999496 | Uncultured ectomycorrhizal fungus | Beech root tip in USA:Ohio |
|  |  |  |  |  |  | 468/481(97%) | JQ711882. | *Tomentella badia*^#^ | Ectomycorrhiza in Canada: BC(52.83 N 123.73 W) |
| 350 | 6 | 20 | Ascomycota | Helotiales | Unassigned | 518/536(97%) | DQ273336 | Uncultured Pezizomycotina | Bulked root pools in USA: California |
|  |  |  |  |  |  | 495/504(98%) | JX001628 | Helotiales sp. | Unreported |
| 351 | 1 | 3 | Basidiomycota | Sporidiobolales | Unassigned | 344/346(99%) | JQ857037 | *Rhodotorula glacialis** | King George Island |
| 352 | 1 | 30 | Rozellomycota | Unassigned | Unassigned | 219/246(89%) | EF635746 | Uncultured fungus (Rozellomycota) | -- |
| 353 | 1 | 3 | Ascomycota | Helotiales | Unassigned | 473/479(99%) | KF297262 | Uncultured fungus | Arctic soil in Canada (76.23N, 119.30W) |
|  |  |  |  |  |  | 452/507(89%) | KF429259 | *Vibrissea* sp. | -- |
| 354 | 4 | 24 | Ascomycota | Helotiales | Unassigned | 503/516(97%) | KF296765 | Uncultured fungus | Arctic soil in Canada (76.23N, 119.30W) |
|  |  |  |  |  |  | 502/541(93%) | FJ378851 | Uncultured Helotiales | -- |
| 355 | 4 | 30 | Ascomycota | Pleosporales | Unassigned | 362/365(99%) | KF617529 | Uncultured fungus | Forest soil in USA: Alaska (63.8138 N 144.9532 W) |
|  |  |  |  |  |  | 368/387(95%) | HQ212254 | Uncultured Pleosporales | -- |
| 356 | 2 | 93 | Ascomycota | Lecanorales | Ramalinaceae | 248/254(98%) | KC965546 | Uncultured fungus | Arctic soil in Canada (76.23N, 119.30W) |
|  |  |  |  |  |  | 227/248 (91%) | AJ247561 | *Biatora tetramera* | -- |
| 357 | 6 | 238 | Ascomycota | Pleosporales | Unassigned | 481/500(96%) | KC965961 | Uncultured fungus | -- |
|  |  |  |  |  |  | 419/468(90%) | FJ839635 | *Mycopappus aceris* | -- |
| 358 | 3 | 8 | Ascomycota | Xylariales | Amphisphaeriaceae | 512/534(96%) | JN871207 | *Seimatosporium walkeri* ^#^ | -- |
| 359 | 6 | 151 | Rozellomycota | Unassigned | Unassigned | 479/494(97%) | KC965937 | Uncultured fungus (Rozellomycota) | Arctic soil in Canada (76.23N, 119.30W) |
| 360 | 7 | 163 | Ascomycota | Chaetothyriales | Herpotrichiellaceae | 429/447(96%) | KC965227 | Uncultured fungus | -- |
|  |  |  |  |  |  | 385/402(96%) | FJ948175 | *Rhinocladiella* sp. ^#^ | -- |
| 361 | 2 | 29 | Unassigned | Unassigned | Unassigned | 462/470(98%) | KF296956 | Uncultured fungus | Arctic soil in Canada (76.23N, 119.30W) |
| 362 | 2 | 20 | Ascomycota | Helotiales | Unassigned | 439/474(93%) | KC965311 | Uncultured fungus | -- |
|  |  |  |  |  |  | 461/509(91%) | JN859274 | Helotiales sp. | -- |
| 363 | 2 | 6 | Basidiomycota | Kriegeriales | Camptobasidiaceae | 409/416(98%) | AY040657 | *Glaciozyma antarctica* ^#^ | Antarctica |
| 364 | 1 | 3 | Ascomycota | Rhizocarpales | Rhizocarpaceae | 469/473(99%) | AF483609 | *Rhizocarpon petraeum** | Norway |
| 365 | 6 | 371 | Ascomycota | Verrucariales | Verrucariaceae | 501/504(99%) | KC966180 | Uncultured fungus | Arctic soil in USA (69.67 N 148.72 W) |
|  |  |  |  |  |  | 435/479(91%) | EU559739 | *Polyblastia* sp. | -- |
| 366 | 5 | 282 | Ascomycota | Pezizales | Pyronemataceae | 439/440(99%) | KF617506 | Uncultured fungus | Arctic soil in USA (69.67 N 148.72 W) |
|  |  |  |  |  |  | 413/415(99%) | JF908022 | *Geopora nicaeensis** | Italy |
| 367 | 2 | 16 | Ascomycota | Sordariales | Lasiosphaeriaceae | 514/521(99%) | JX489802 | Uncultured soil fungus | Soil in China: Heilongjiang, Harbin |
|  |  |  |  |  |  | 491/499(98%) | GU05573 | Uncultured *Schizothecium* ^#^ | Agricultural soil in Austria: Tulln（[48.33 N 16.05 E](http://www.ncbi.nlm.nih.gov/projects/Sequin/latlonview.html?lat=48.33333&lon=16.05000)） |
| 368 | 4 | 74 | Ascomycota | Capnodiales | Teratosphaeriaceae | 490/514(95%) | KF296855 | Uncultured fungus | -- |
|  |  |  |  |  |  | 443/469(94%) | KF309972 | *Oleoguttula mirabilis* | -- |
| 369 | 7 | 622 | Ascomycota | Chaetothyriales | Herpotrichiellaceae | 430/439(98%) | KC965460 | Uncultured fungus | Arctic soil in Canada (76.23N, 119.30W) |
|  |  |  |  |  |  | 380/394(96%) | FJ948175 | *Rhinocladiella* sp. ^#^ | -- |
| 370 | 1 | 10 | Ascomycota | Xylariales | Amphisphaeriaceae | 515/522(99%) | KT162918 | *Seimatosporium cornii** | Branch in Italy |
| 371 | 1 | 4 | Unassigned | Unassigned | Unassigned | 323/395(82%) | KF296936 | Uncultured fungus | -- |
| 372 | 2 | 31 | Glomeromycota | Unassigned | Unassigned | 340/366(93%) | KF296911 | Uncultured fungus | -- |
|  |  |  |  |  |  | 289/334(93%) | HQ211979 | Uncultured Glomeromycota | -- |
| 373 | 6 | 157 | Ascomycota | Verrucariales | Unassigned | 486/528(92%) | KC965806 | Uncultured fungus | -- |
|  |  |  |  |  |  | 453/509(89%) | FJ664852 | *Verrucaria* sp. | -- |
| 374 | 1 | 10 | Ascomycota | Hypocreales | Unassigned | 405/406(99%) | KM580048 | *Acremonium implicatum** | Roots in South Africa (33.19 S 26.31 E) |
| 375 | 1 | 18 | Ascomycota | Teloschistales | Teloschistaceae | 430/430(100%) | KC966222 | Uncultured fungus | Arctic soil in Canada (78.78 N 103.55 W) |
|  |  |  |  |  |  | 384/384 (100%) | KC179111 | *Parvoplaca athallina** | Antarctica |
| 376 | 5 | 287 | Ascomycota | Lecideales | Lecideaceae | 320/357(90%) | AM292669 | *Bilimbia microcarpa* | -- |
| 377 | 5 | 64 | Ascomycota | Pleosporales | Sporomiaceae | 527/529(99%) | KP698359 | Ascomycota sp. | Dehesa ecosystem in Spain (38.45 N 6.98 W) |
|  |  |  |  |  |  | 509/512(99%) | HQ602666 | *Preussia* sp. ^#^ | Sterilized needle tissue of *Pinus monticola* in USA |
| 378 | 6 | 232 | Ascomycota | Pleosporales | Unassigned | 467/485(96%) | KC966086 | Uncultured fungus | -- |
|  |  |  |  |  |  | 444/509(87%) | HQ631002 | Pleosporales sp. | -- |
| 379 | 1 | 13 | Ascomycota | Helotiales | Unassigend | 453/520(87%) | KF617563 | Uncultured fungus | -- |
|  |  |  |  |  |  | 283/297(95%) | KM113762 | Helotiales sp. | -- |
| 380 | 1 | 13 | Ascomycota | Hypocreales | Nectriaceae | 403/406(99%) | JX388038 | Uncultured fungus | Soil in USA |
|  |  |  |  |  |  | 402/405(99%) | JN207347 | *Fusarium* sp. ^#^ | Plant in Northwest Venezula |
| 381 | 1 | 36 | Glomeromycota | Unassigned | Unassigned | 421/485(87%) | KF296911 | Uncultured fungus | -- |
|  |  |  |  |  |  | 336/405 (83%) | HQ211979 | Uncultured Glomeromycota | -- |
| 382 | 1 | 4 | Basidiomycota | Unassigned | Unassigned | 268/316(85%) | JQ666673 | Uncultured soil fungus | -- |
|  |  |  |  |  |  | 258/316(84%) | [HM240148](http://www.ncbi.nlm.nih.gov/nucleotide/170516703?report=genbank&log$=nuclalign&blast_rank=2&RID=2GWT06BX013) | Uncultured Basidiomycota | -- |
| 383 | 5 | 97 | Ascomycota | Umbilicariales | Unassigned | 423/482(88%) | AF096214 | *Umbilicaria decussata* | -- |
| 384 | 3 | 9 | Basidiomycota | Agaricales | Cortinariaceae | 495/497(99%) | KP889761 | Uncultured fungus | soil |
|  |  |  |  |  |  | 494/496(99%) | JQ724020 | *Cortinarius diasemospermus** | Ectomycorrhiza in Sweden (59.49 N 17.40 E) |
| 385 | 2 | 6 | Ascomycota | Chaetothyriales | Herpotrichiellaceae | 384/409(94%) | KP889575 | Uncultured fungus | -- |
|  |  |  |  |  |  | 377/405(93%) | FJ553220 | Uncultured Herpotrichiellaceae | -- |
| 386 | 2 | 39 | Ascomycota | Pleosporales | Unassigned | 503/506(99%) | KC966092 | Uncultured fungus | Arctic soil in Canada (76.23N, 119.30W) |
|  |  |  |  |  |  | 518/533(97%) | JX010732 | Uncultured *Phoma* ^#^ | Plant (*Populus euphratica*) in China: Yuli county |
| 387 | 3 | 96 | Ascomycota | Helotiales | Unassigned | 464/494(94%) | KC965313 | Uncultured fungus | -- |
|  |  |  |  |  |  | 463/518(89%) | KM113762 | Helotiales sp. | -- |
| 388 | 1 | 5 | Ascomycota | Capnodiales | Mycosphaerellaceae | 508/526(97%) | KJ504803 | *Ramularia miae* ^#^ | *Wachendorfia thyrsiflora* in South Africa |
| 389 | 4 | 16 | Ascomycota | Pleosporales | Phaeosphaeriaceae | 499/508(98%) | FJ820772 | Uncultured fungus | Air sample in Germany |
|  |  |  |  |  |  | 452/462 (98%) | AF439488 | *Phaeosphaeria juncophila** | Unreported |
| 390 | 8 | 390 | Ascomycota | Hypocreales | Nectriaceae | 511/512(99%) | KP889860 | Uncultured fungus | Soil in forest |
|  |  |  |  |  |  | 510/512 (99%) | GU055710 | Uncultured *Nectria* ^#^ | Grassland soil in Austria: Riederberg (48.25N 16.06E) |
| 391 | 3 | 31 | Unassigned | Unassigned | Unassigned | 323/365(88%) | JX330945 | Uncultured fungus | -- |
| 392 | 3 | 258 | Ascomycota | Capnodiales | Unassigned | 488/491(99%) | KC965419 | Uncultured fungus | Arctic soil in USA (69.67 N 148.72 W) |
|  |  |  |  |  |  | 347/406 (85%) | HQ599590 | *Pseudocercospora nephrolepidicola* | -- |
| 393 | 1 | 4 | Ascomycota | Hypocreales | Unassigned | 344/358(96%) | KC965583 | Uncultured fungus | -- |
|  |  |  |  |  |  | 346/383(90%) | FR799492 | *Acremonium* sp. | -- |
| 394 | 2 | 14 | Ascomycota | Chaetothyriales | Herpotrichiellaceae | 346/350(99%) | EF635808 | Uncultured fungus | Soil beneath *Salix herbacea* in Austria (46.83 N 11.05 E) |
|  |  |  |  |  |  | 410/449(91%) | JF747078 | *Exophiala* sp. | -- |
| 395 | 1 | 64 | Ascomycota | Pleosporales | Unassigned | 447/447(100%) | JQ759518 | Dothideomycetes sp. | Photosynthetic tissue in USA: AK(64.5011 N 165.4064 W) |
|  |  |  |  |  |  | 495/571(87%) | FJ475806 | Uncultured Pleosporales | -- |
| 396 | 5 | 79 | Ascomycota | Helotiales | Unassigned | 534/537(99%) | FJ554342 | Uncultured Ascomycota | Forest soil in Canada |
|  |  |  |  |  |  | 519/537(97%) | GU327472 | Uncultured *Tetracladium* ^#^ | Mycorrhizal seedling in Czech |
| 397 | 1 | 4 | Chytridiomycota | Unassigned | Unassigned | 365/368(99%) | JX367564 | Uncultured fungus | Soil in USA |
|  |  |  |  |  |  | 327/382(86%) | EU873019 | Uncultured Chytridiomycota . | -- |
| 398 | 7 | 109 | Ascomycota | Hypocreales | Ophiocordycipitaceae | 532/537(99%) | AB208110 | *Tolypocladium cylindrosporum** | Unreported |
| 399 | 2 | 20 | Glomeromycota | Unassigned | Unassigned | 355/375(95%) | KF297175 | Uncultured fungus | -- |
|  |  |  |  |  |  | 183/203(90%) | EF619905 | Uncultured Glomeromycota | -- |
| 400 | 1 | 38 | Ascomycota | Capnodiales | Teratosphaeriaceae | 494/532(93%) | HM240000 | Uncultured Ascomycota | -- |
|  |  |  |  |  |  | 494/532(93%) | JF499843 | *Penidiella ellipsoidea* | -- |
| 401 | 1 | 25 | Basidiomycota | Tremellales | Unassigned | 343/366(94%) | KC966086 | Uncultured fungus | -- |
|  |  |  |  |  |  | 289/291(99%) | KP299250 | *Cryptococcus victoriae** | Antarctic snow |
| 402 | 7 | 76 | Ascomycota | Helotiales | Unassigned | 493/507(97%) | JX359581 | Uncultured fungus | Soil in USA |
|  |  |  |  |  |  | 459/488(94%) | KR063494 | Uncultured *Tetracladium* | -- |
| 403 | 8 | 1522 | Ascomycota | Helotiales | Unassigned | 513/520(99%) | KC965311 | Uncultured fungus | Arctic soil in Canada (73.22N, 119.56W) |
|  |  |  |  |  |  | 514/546(94%) | KC694156 | Uncultured *Tetracladium* | -- |
| 404 | 1 | 141 | Ascomycota | Helotiales | Helotiaceae | 479/487(98%) | KC965767 | Uncultured fungus | Arctic soil in Canada (78.78 N 103.55 W) |
|  |  |  |  |  |  | 477/510(94%) | FJ553703 | *Claussenomyces* sp. | -- |
| 405 | 7 | 123 | Zygomycota | Mortierellales | Mortierellaceae | 492/500(98%) | GQ219843 | Uncultured Mortierellaceae | Soil in Germany: Thuringia, Hainich |
|  |  |  |  |  |  | 456/464(98%) | KP411578 | *Mortierella polygonia* ^#^ | Soil in the caves of Glacier National Park (Canada) |
| 406 | 7 | 113 | Ascomycota | Helotiales | Unassigend | 493/500(99%) | KC965944 | Uncultured fungus | Arctic soil in Canada (73.22N, 119.56W) |
|  |  |  |  |  |  | 387/413(93%) | HQ845751 | Helotiales sp. | -- |
| 407 | 1 | 21 | Basidiomycota | Sebacinales | Sebacinaceae | 493/501(98%) | GQ907094 | Uncultured *Sebacina* ^#^ | In United Kingdom: Scotland |
| 408 | 1 | 2 | Ascomycota | Unassigned | Unassigned | 374/447(84%) | EU263928 | *Lecidea* sp. | -- |
| 409 | 22 | 204 | Ascomycota | Helotiales | Unassigend | 485/493(98%) | KF296739 | Uncultured fungus | Arctic soil in Canada (76.23N, 119.30W) |
|  |  |  |  |  |  | 465/490(94%) | JN859275 | Helotiales sp. | -- |
| 410 | 3 | 22 | Ascomycota | Helotiales | Unassigned | 397/414(96%) | JX381513 | Uncultured fungus | -- |
|  |  |  |  |  |  | 394/414(95%) | KR063523 | Uncultured Helotiales | -- |
| 411 | 1 | 9 | Chytridiomycota | Olpidiales | Olpidiaceae | 335/346(97%) | KF493955 | Uncultured *Olpidium* ^#^ | Roots of tomato grown on farm soil |
| 412 | 1 | 5 | Basidiomycota | Thelephorales | Thelephoraceae | 457/474(96%) | FJ581422 | Uncultured *Tomentella* ^#^ | -- |
| 413 | 2 | 34 | Ascomycota | Verrucariales | Verrucariaceae | 507/510(99%) | KF296962 | Uncultured fungus | Arctic soil in Canada (76.23N, 119.30W) |
|  |  |  |  |  |  | 459/498(92%) | JF509161 | *Agonimia repleta* | -- |
| 414 | 3 | 77 | Basidiomycota | Thelephorales | Thelephoraceae | 464/465(99%) | KF618003 | Uncultured fungus | Forest soil in USA: Alaska (64.86 N 147.87 W) |
|  |  |  |  |  |  | 463/466 (99%) | JX630521 | Uncultured *Tomentella* ^#^ | Plant root in Greenland (76.53N, 68.76W) |
| 415 | 1 | 10 | Basidiomycota | Auriculariales | Auriculariaceae | 332/343(97%) | FJ237097 | Uncultured fungus | Snow covered alpine soil in Austria (glacier foreland) |
|  |  |  |  |  |  | 224/235 (95%) | JX065165 | *Auricularia delicata* ^#^ | -- |
| 416 | 1 | 15 | Ascomycota | Helotiales | Unassigned | 355/365(97%) | KC965311 | Uncultured fungus | Arctic soil in Canada (73.22N, 119.56W) |
|  |  |  |  |  |  | 360/387(93%) | GU327472 | Uncultured *Tetracladium* | -- |
| 417 | 1 | 13 | Chytridiomycota | Chytridiales | Unassigned | 334/409(82%) | JX364856 | Uncultured fungus | -- |
|  |  |  |  |  |  | 260/280(92%) | EU352773 | Chytridiales sp. | -- |
| 418 | 2 | 13 | Ascomycota | Capnodiales | Teratosphaeriaceae | 350/376(93%) | KF296855 | Uncultured fungus | -- |
|  |  |  |  |  |  | 293/315 (93%) | KF309972 | *Oleoguttula mirabilis* | -- |
| 419 | 1 | 238 | Chytridiomycota | Rhizophydiales | Unassigned | 328/406(81%) | KF296964 | Uncultured fungus | -- |
|  |  |  |  |  |  | 219/219 (100%) | EF634250 | Rhizophydiales sp. | Unreported |
| 420 | 1 | 49 | Basidiomycota | Sebacinales | Sebacinaceae | 466/510(91%) | KC986274 | Uncultured *Sebacina* | -- |
| 421 | 3 | 119 | Glomeromycota | Unassigned | Unassigned | 453/455(99%) | KF296911 | Uncultured fungus | Arctic soi in Canada (73.22N, 119.56W) |
|  |  |  |  |  |  | 344/390(88%) | GU392007 | Uncultured Glomeromycota | -- |
| 422 | 1 | 7 | Ascomycota | Xylariales | Amphisphaeriaceae | 371/377(98%) | FR773218 | Uncultured Amphisphaeriaceae | Unreported |
| 423 | 2 | 10 | Ascomycota | Helotiales | Unassigned | 392/399(98%) | KF617342 | Uncultured fungus | Foest soil in USA (Alaska, 64.91N, 147.82W) |
|  |  |  |  |  |  | 356/382(93%) | FJ827178 | Uncultured Helotiales | -- |
| 424 | 9 | 330 | Ascomycota | Helotiales | Unassigned | 506/516(98%) | FJ554384 | Uncultured Leotiomycetes | Forest soil in Canada |
|  |  |  |  |  |  | 448/456(98%) | KM216321 | Helotiales sp. | Needles of *Pinus radiata* in Tasmania |
| 425 | 7 | 841 | Ascomycota | Helotiales | Unassigned | 517/530(98%) | JQ666656 | Uncultured fungus | Forest soil in China (Changbai mountain) |
|  |  |  |  |  |  | 490/492(99%) | FJ553913 | Uncultured *Tetracladium* ^#^ | Forest soil in Canada |
| 426 | 1 | 36 | Ascomycota | Unassigned | Unassigned | 307/373(82%) | HM537043 | Fungal endophyte sp. | -- |
|  |  |  |  |  |  | 307/374(82%) | GQ469961 | *Lasiodiplodia parva* | -- |
| 427 | 7 | 117 | Ascomycota | Unassigned | Unassigned | 367/380(97%) | KF296938 | Uncultured fungus | Arctic soil in Canada (76.23N, 119.30W) |
|  |  |  |  |  |  | 454/538(84%) | FJ553946 | Uncultured Herpotrichiellaceae | -- |
| 428 | 2 | 3 | Ascomycota | Helotiales | Unassigend | 476/492(97%) | HG327912 | Uncultured fungus | Arable soil in China (Gongzhuling) |
|  |  |  |  |  |  | 461/483(95%) | JN859275 | Helotiales sp. | -- |
| 429 | 1 | 20 | Basidiomycota | Tremellales | Tremellaceae | 259/263(98%) | JN906687 | Uncultured fungus | Plant phyllosphaere in France |
|  |  |  |  |  |  | 264/290(91%) | KP691956 | *Bullera* sp. | -- |
| 430 | 1 | 6 | Ascomycota | Unassigned | Unassigned | 402/409(98%) | KC966180 | Uncultured fungus (Ascomycota) | Arctic soil in USA (69.67N, 148.72W) |
| 431 | 2 | 15 | Ascomycota | Pleosporales | Unassigned | 278/282(99%) | JN906690 | Uncultured fungus | Plant phyllosphaere in France |
|  |  |  |  |  |  | 232/238(97%) | EU516991 | Uncultured fungus (Pleosporales) | -- |
| 432 | 6 | 493 | Ascomycota | Verrucariales | Unassigned | 462/496(93%) | KC965806 | Uncultured fungus | -- |
|  |  |  |  |  |  | 451/505(89%) | FJ664852 | *Verrucaria* sp. | -- |
| 433 | 5 | 148 | Ascomycota | Chaetothyriales | Herpotrichiellaceae | 564/582(97%) | KC965460 | Uncultured fungus | Arctic soil in Canada (76.23N, 119.30W) |
|  |  |  |  |  |  | 498/520(96%) | FJ948175 | *Rhinocladiella* sp. ^#^ | -- |
| 434 | 2 | 20 | Ascomycota | Helotiales | Unassigned | 488/522(93%) | HQ845751 | Helotiales sp. | -- |
| 435 | 2 | 19 | Ascomycota | Helotiales | Unassigned | 512/525(98%) | FJ554282 | Uncultured fungus | Forest soil in Canada |
|  |  |  |  |  |  | 494/504(98%) | KC455325 | Uncultured Helotiales | Plant root in USA (Alaska) |
| 436 | 1 | 2 | Ascomycota | Verrucariales | Verrucariaceae | 289/292(99%) | KC966180 | Uncultured fungus | Arctic soil in USA (69.67N, 148.72W) |
|  |  |  |  |  |  | 285/302(94%) | FJ664858 | *Verrucaria* sp. | -- |
| 437 | 2 | 40 | Ascomycota | Lecideales | Lecideaceae | 496/527(94%) | KC965546 | Uncultured fungus | -- |
|  |  |  |  |  |  | 415/449(92%) | AM292706 | *Mycobilimbia tetramera* | -- |
| 438 | 2 | 32 | Ascomycota | Helotiales | Unassigned | 345/368(94%) | EU292563 | Uncultured fungus | -- |
|  |  |  |  |  |  | 282/302(93%) | KC965638 | Uncultured fungus (*Variosporium*) |  |
| 439 | 1 | 3 | Basidiomycota | Cantharellales | Clavulinaceae | 357/358(99%) | HQ444982 | Uncultured fungus | Plant root in alpine and Arctic |
|  |  |  |  |  |  | 386/424(91%) | KF218966 | *Membranomyces spurius* | -- |
| 440 | 4 | 13 | Ascomycota | Pleosporales | Unassigned | 518/520(99%) | JX984777 | Uncultured fungus | TSP in urban air in Seoul |
|  |  |  |  |  |  | 492/520(95%) | EU167561 | *Pleiochaeta ghindensis* ^#^ | -- |
| 441 | 1 | 9 | Ascomycota | Helotiales | Leotiaceae | 510/521(98%) | KC834041 | *Alatospora flagellata* ^#^ | Stream, *Fagus sylvatica* leaf, CZ |
| 442 | 1 | 4 | Ascomycota | Verrucariales | Verrucariaceae | 287/288(99%) | KC965643 | Uncultured fungus | Arctic soil in Canada (76.23N, 119.30W) |
|  |  |  |  |  |  | 271/299(91%) | KM243179 | *Verrucaria alpicola* | -- |
| 443 | 4 | 264 | Ascomycota | Lecanorales | Porpidiaceae | 506/515(98%) | KC965546 | Uncultured fungus | Arctic soil in Canada (76.23N, 119.30W) |
|  |  |  |  |  |  | 480/510(94%) | AJ247561 | *Biatora tetramera* | -- |
| 444 | 2 | 129 | Ascomycota | Unassigned | Unassigned | 502/538(93%) | KF297139 | Uncultured fungus | -- |
|  |  |  |  |  |  | 253/272(92%) | JF683420 | *Hypoderma cordylines* | -- |
| 445 | 5 | 42 | Ascomycota | Capnodiales | Teratosphaeriaceae | 500/517(97%) | KC966025 | Uncultured fungus | Arctic soil in Canada (76.23N, 119.30W) |
|  |  |  |  |  |  | 448/466(96%) | KF309972 | *Oleoguttula mirabilis* ^#^ | -- |
| 446 | 2 | 5 | Ascomycota | Chaetothyriales | Herpotrichiellaceae | 376/389(97%) | KF297101 | Uncultured fungus | Arctic soil in USA (70.31N, 147.99W) |
|  |  |  |  |  |  | 385/423(91%) | FJ554329 | Uncultured Herpotrichiellaceae | -- |
| 447 | 4 | 14 | Ascomycota | Umbilicariales | Unassigned | 281/321(88%) | AJ431598 | *Umbilicaria kappeni* | -- |
| 448 | 1 | 163 | Ascomycota | Helotiales | Unassigned | 500/505(99%) | KC965448 | Uncultured fungus | Arctic soil in Canada (76.23N, 119.30W) |
|  |  |  |  |  |  | 472/535(88%) | HQ845751 | Helotiales sp. | -- |
| 449 | 1 | 4 | Ascomycota | Phacidiales | Unassigned | 321/371(87%) | HQ701751 | Uncultured fungus | -- |
|  |  |  |  |  |  | 317/368(86%) | KM108373 | *Darkera picea* | -- |
| 450 | 1 | 10 | Ascomycota | Pezizales | Tuberaceae | 428/445(96%) | KF296834 | Uncultured fungus | -- |
|  |  |  |  |  |  | 428/445(96%) | JX630610 | Uncultured *Choiromyces* ^#^ | -- |
| 451 | 1 | 10 | Ascomycota | Lecanorales | Unassigned | 394/451(87%) | AY425668 | *Protoblastenia cyclospora* | -- |
| 452 | 1 | 10 | Basidiomycota | Unassigned | Unassigned | 325/377(86%) | AM901823 | Uncultured basidiomycete | -- |
| 453 | 3 | 8 | Ascomycota | Hypocreales | Unassigned | 422/433(97%) | KP889967 | Uncultured fungus | Forest soil in Canada |
|  |  |  |  |  |  | 421/433(97%) | HQ631053 | *Stilbella* sp. ^#^ | Plant (*Saccharum officinarum*) |
| 454 | 2 | 31 | Ascomycota | Sordariales | Lasiosphaeriaceae | 412/426(97%) | JX358195 | Uncultured fungus | Soil in USA |
|  |  |  |  |  |  | 387/402 (96%) | AY999118 | *Schizothecium carpinicola* ^#^ | -- |
| 455 | 1 | 14 | Basidiomycota | Agaricales | Cortinariaceae | 202/203(99%) | FJ554223 | Uncultured *Cortinarius* ^#^ | Forest soil in Canada |
|  |  |  |  |  |  | 201/202(99%) | JQ711860 | *Cortinarius* sp. ^#^ | Ectomycorrhiza in Canada (52.83N, 123.73W) |
| 456 | 8 | 749 | Ascomycota | Umbilicariales | Unassigned | 495/502(99%) | KF296938 | Uncultured fungus | Arctic soil in Canada (76.23N, 119.30W) |
|  |  |  |  |  |  | 415/485(86%) | AF096216 | *Umbilicaria hyperborea* | -- |
| 457 | 1 | 2 | Ascomcyota | Helotiales | Unassigned | 298/315(95%) | KC966260 | Uncultured fungus | -- |
|  |  |  |  |  |  | 297/337(88%) | FJ553913 | Uncultured *Tetracladium* | -- |
| 458 | 2 | 6 | Ascomycota | Unassigned | Unassigned | 396/485(82%) | EU490139 | Uncultured Ascomycota | -- |
| 459 | 3 | 35 | Ascomycota | Pleosporales | Unassigned | 480/490(98%) | KC966076 | Uncultured fungus | Soil in Canada (76.23N, 119.30W) |
|  |  |  |  |  |  | 485/510(95%) | FJ552958 | Uncultured Pleosporales | -- |
| 460 | 1 | 9 | Ascomycota | Capnodiales | Teratosphaeriaceae | 474/490(97%) | AM901736 | Uncultured ascomycete | House dust in Finland |
|  |  |  |  |  |  | 460/492(93%) | GU570527 | *Devriesia pseudoamericana* | - |
| 461 | 4 | 34 | Ascomycota | Helotiales | Unassigned | 435/462(94%) | FJ554342 | Uncultured Ascomycota | Forest soil in Canada |
|  |  |  |  |  |  | 387/396(98%) | JX029114 | *Tetracladium* *psychrophilum* ^#^ | Qinghai-Tibet plateau |
| 462 | 1 | 43 | Ascomycota | Verrucariales | Verrucariaceae | 338/372(91%) | FJ664859 | *Verrucaria* sp. | -- |
| 463 | 2 | 181 | Chytridiomycota | Unassigned | Unassigned | 506/594(85%) | KC965876 | Uncultured fungus | -- |
|  |  |  |  |  |  | 296/356(83%) | HQ191313 | Uncultured Chytridiomycota | Lake in France (45.50N 2.88E) |
| 464 | 2 | 8 | Unassigned | Unassigned | Unassigned | 323/333(97%) | FJ197888 | Uncultured fungus | Primary successional glacier foreland soil |
| 465 | 1 | 11 | Basidiomycota | Agaricales | Inocybaceae | 418/430(97%) | JF908117 | *Inocybe geraniodora* ^#^ | Italy |
| 466 | 1 | 2 | Rozellomycota | Unassigned | Unassigned | 284/317(90%) | KC965607 | Uncultured fungus | -- |
|  |  |  |  |  |  | 270/318(85%) | EF521235 | Uncultured fungus (Rozellomycota) | -- |
| 467 | 1 | 20 | Chytridiomycota | Unassigned | Unassigned | 310/360(86%) | GU065483 | Uncultured fungus | -- |
|  |  |  |  |  |  | 390/486(80%) | HQ191313 | Uncultured Chytridiomycota | -- |
| 468 | 1 | 24 | Ascomycota | Orbiliales | Orbiliaceae | 446/508(88%) | KF296869 | Uncultured fungus (Orbiliaceae) | -- |
|  |  |  |  |  |  | 401/502(80%) | HM161962 | Uncultured Ascomycota | -- |
| 469 | 5 | 39 | Ascomycota | Pleosporales | Phaeosphaeriaceae | 515/527(98%) | AM901822 | Uncultured ascomycete | House dust in Finland |
|  |  |  |  |  |  | 460/467(99%) | AF439507 | *Phaeosphaeria triglochinicola* * | Plant in Switzerland |
| 470 | 1 | 6 | Ascomycota | Teloschistales | Physciaceae | 424/426(99%) | KC965134 | Uncultured fungus | Arctic soil in Canada (76.23N, 119.30W) |
|  |  |  |  |  |  | 366/406(90%) | AF278752 | *Phaeophyscia* sp. | -- |
| 471 | 1 | 4 | Ascomycota | Pleosporales | Phaeosphaeriaceae | 405/428(95%) | KF800344 | Uncultured fungus | House dust in USA (Missouri, Kansas) |
|  |  |  |  |  |  | 405/429(94%) | JX517283 | *Sclerostagonospora* sp. | -- |
| 472 | 1 | 8 | Ascomycota | Lecanorales | Unassigned | 484/489(99%) | KF297261 | Uncultured fungus | Arctic soil in Canada (76.23N, 119.30W) |
|  |  |  |  |  |  | 390/456(86%) | HQ650647 | *Squamarina gypsacea* | -- |
| 473 | 4 | 130 | Ascomycota | Capnodiales | Teratosphaeriaceae | 464/487(95%) | JQ247386 | Uncultured fungus | -- |
|  |  |  |  |  |  | 482/520(93%) | JF499843 | *Penidiella ellipsoidea* | -- |
| 474 | 1 | 5 | Ascomycota | Helotiales | Unassigned | 489/501(98%) | KC694158 | Uncultured Ascomycota | Plant roots in Sweden |
|  |  |  |  |  |  | 417/440(95%) | JN581127 | *Tetracladium* sp. ^#^ | -- |
| 475 | 3 | 37 | Chytridiomycota | Chytridiales | Unassigned | 270/319(85%) | JX344420 | Uncultured fungus | -- |
|  |  |  |  |  |  | 231/247(93%) | EU352774 | Chytridiales sp. | -- |
| 476 | 1 | 784 | Ascomycota | Lecanorales | Unassigned | 503/509(99%) | KC965500 | Uncultured fungus | Arctic soil in USA (69.67N, 148.72W) |
|  |  |  |  |  |  | 210/234(90%) | AF282094 | *Bacidina inundata* | -- |
| 477 | 2 | 29 | Unassigned | Unassigned | Unassigned | 188/203(93%) | HM069466 | Uncultured fungus | -- |
| 478 | 2 | 19 | Chytridiomycota | Chytridiales | Chytriomycetaceae | 346/384(90%) | GU065483 | Uncultured fungus | -- |
|  |  |  |  |  |  | 257/269(96%) | AY997049 | *Entophlyctis* sp. ^#^ | -- |
| 479 | 5 | 11 | Rozellomycota | Unassigned | Unassigned | 449/466(96%) | KP889857 | Uncultured fungus | -- |
|  |  |  |  |  |  | 355/384(92%) | EF635746 | Uncultured fungus (Rozellomyccota) |  |
| 480 | 3 | 8 | Ascomycota | Saccharomycetales | Pichiaceae | 354/355(99%) | FR839631 | *Pichia pastoris** | unreported |
| 481 | 5 | 1884 | Ascomycota | Helotiales | Unassigned | 446/460(97%) | KF617412 | Uncultured fungus | Forest soil in USA (64.86N, 147.87W) |
|  |  |  |  |  |  | 470/513(92%) | KM113762 | Helotiales sp. | -- |
| 482 | 2 | 9 | Rozellomycota | Unassigned | Unassigned | 331/341(97%) | AF504875 | Uncultured fungus (Rozellomycota) | Grassland soil in Scotland, UK |
| 483 | 1 | 17 | Ascomycota | Chaetothyriales | Unassigned | 478/537(89%) | KC965841 | Uncultured fungus | -- |
|  |  |  |  |  |  | 415/476(87%) | KP400573 | Chaetothyriales sp. | -- |
| 484 | 1 | 11 | Chytridiomycota | Rhizophydiales | Unassigned | 385/411(94%) | KF296736 | Uncultured fungus | -- |
|  |  |  |  |  |  | 233/259(89%) | EF585656 | *Boothiomyces* sp. | -- |
| 485 | 3 | 100 | Ascomycota | Saccharomycetales | Unassigned | 436/467(93%) | KF297139 | Uncultured fungus | -- |
|  |  |  |  |  |  | 181/200(91%) | JN183452 | *Candida bituminiphila* | -- |
| 486 | 1 | 5 | Ascomycota | Verrucariales | Verrucariaceae | 443/449(99%) | KF297283 | Uncultured fungus | Arctic soil in Canada (73.22N, 119.56W) |
|  |  |  |  |  |  | 391/435(90%) | FJ664852 | *Verrucaria* sp. | -- |
| 487 | 2 | 35 | Ascomycota | Pezizales | Pyronemataceae | 470/481(98%) | KF617360 | Uncultured fungus | Forest soil in USA (64.91N, 147.82W) |
|  |  |  |  |  |  | 468/481(97%) | JX630356 | Uncultured Pyronemataceae | Plant root in USA (70.30N, 147.98W) |
| 488 | 1 | 12 | Chytridiomycota | Unassigned | Unassigned | 404/464(87%) | KC965876 | Uncultured fungus | -- |
|  |  |  |  |  |  | 286/355(81%) | HQ191313 | Uncultured Chytridiomycota | -- |
| 489 | 2 | 13 | Ascomycota | Helotiales | Unassigned | 497/498(99%) | KC965466 | Uncultured fungus | Arctic soil in Canada (73.22N, 119.56W) |
|  |  |  |  |  |  | 495/519(95%) | FJ378851 | Uncultured Helotiales | -- |
| 490 | 2 | 15 | Glomeromycota | Unassigned | Unassigned | 478/486(98%) | JQ666442 | Uncultured soil fungus | Forest soil in China (Changbai Mountain) |
|  |  |  |  |  |  | 387/418(98%) | EF619906 | Uncultured Glomeromycota | Forest soil in USA (Orange Co., NC) |
| 491 | 3 | 82 | Ascomycota | Helotiales | Unassigned | 495/497(99%) | KC965195 | Uncultured fungus | Arctic soil in USA (69.67N, 148.72W) |
|  |  |  |  |  |  | 497/523(95%) | JN859274 | Helotiales sp. | -- |
| 492 | 1 | 39 | Basidiomycota | Agaricales | Inocybaceae | 479/481(99%) | FJ378759 | Uncultured *Inocybe* ^#^ | Ectomycorrhiza in the eastern Himalaya |
| 493 | 2 | 41 | Ascomycota | Helotiales | Hyaloscyphaceae | 516/525(98%) | DQ420921 | Uncultured soil fungus | Soil in USA |
|  |  |  |  |  |  | 515/525(98%) | FJ378855 | Uncultured *Lachnum* | Ectomycorrhiza in an alpine meadow in the Himalaya |
| 494 | 2 | 48 | Ascomycota | Pleosporales | Sporomiaceae | 474/490(97%) | JX984761 | Uncultured fungus | TSP in urban air in Seoul |
|  |  |  |  |  |  | 468/482(97%) | HQ602666 | *Preussia* sp. ^#^ | Surface sterilized needle tissue in USA |
| 495 | 3 | 9 | Basidiomycota | Agaricales | Cortinariaceae | 398/402(99%) | EU668257 | Uncultured *Cortinarius* ^#^ | Mycorrhizal plant roots in forest soil (Wales) |
| 496 | 2 | 5 | Ascomycota | Eurotiales | Trichocomaceae | 405/405(100%) | JX984696 | Uncultured fungus | TSP in urban air in Seoul |
|  |  |  |  |  |  | 404/405(99%) | HM991283 | *Eurotium* sp. ^#^ | Gorgonian in China (South China Sea) |
| 497 | 1 | 110 | Ascomycota | Pezizales | Pezizaceae | 514/522(98%) | AJ875374 | Uncultured fungus | Plant in Germany |
|  |  |  |  |  |  | 488/509(96%) | JF908544 | *Peziza badia* ^#^ | -- |
| 498 | 2 | 6 | Ascomycota | Helotiales | Unassigned | 490/513(96%) | KC965753 | Uncultured fungus | -- |
|  |  |  |  |  |  | 480/533(90%) | FJ553913 | Uncultured *Tetracladium* | -- |
| 499 | 5 | 164 | Ascomycota | Verrucariales | Verrucariaceae | 475/498(95%) | KC966180 | Uncultured fungus | -- |
|  |  |  |  |  |  | 429/479(90%) | EU559739 | *Polyblastia* sp. | -- |
| 500 | 1 | 9 | Ascomycota | Pleosporales | Unassigned | 451/519(87%) | HQ631052 | Pleosporales sp. | -- |
| 501 | 2 | 15 | Ascomycota | Helotiales | Unassigned | 449/494(91%) | KC966277 | Uncultured fungus | -- |
|  |  |  |  |  |  | 361/383(94%) | KF730848 | *Varicosporium delicatum* | -- |
| 502 | 1 | 20 | Unassigned | Unassigned | Unassigned | 370/414(89%) | KC966235 | Uncultured fungus | -- |
| 503 | 1 | 7 | Unassigned | Unassigned | Unassigned | 355/390(91%) | KP889925 | Uncultured fungus | -- |
| 504 | 4 | 17 | Basidiomycota | Agaricales | Tricholomataceae | 474/481(99%) | GU234093 | *Omphalina rustica** | Svalbard |
| 505 | 3 | 6 | Basidiomycota | Tremellales | Trichosporonaceae | 428/441(97%) | GQ219946 | Uncultured fungus | Soil in Germany |
|  |  |  |  |  |  | 426/441(97%) | JX270363 | *Trichosporon* sp. ^#^ | Soil from bat hibernaculum in USA |
| 506 | 6 | 31 | Ascomycota | Helotiales | Unassigned | 401/412(97%) | JX349824 | Uncultured fungus | Soil |
|  |  |  |  |  |  | 363/371(97%) | KC694157 | Uncultured *Tetracladium* ^#^ | Plant root in Sweden |
| 507 | 1 | 27 | Ascomycota | Lecanorales | Ramalinaceae | 483/498(97%) | JN972444 | *Bacidina neosquamulosa* ^#^ | Slovakia |
| 508 | 3 | 358 | Ascomycota | Coniochaetales | Coniochaetaceae | 531/538(99%) | FJ552766 | Uncultured Sordariomycetes | Forest soil in Canada |
|  |  |  |  |  |  | 508/544(93%) | KJ957775 | *Coniochaeta* sp. | -- |
| 509 | 1 | 16 | Glomeromycota | Unassigned | Unassigned | 301/330(91%) | FJ197925 | Uncultured fungus | -- |
|  |  |  |  |  |  | 285/327(87%) | GU392007 | Uncultured Glomeromycota | -- |
| 510 | 2 | 7 | Basidiomycota | Agaricales | Strophariaceae | 404/413(98%) | GU234139 | *Alnicola tantilla* ^#^ | Svalbard |
| 511 | 1 | 16 | Chytridiomycota | Unassigned | Unassigned | 303/364(82%) | HQ191313 | Uncultured Chytridiomycota | -- |
| 512 | 1 | 8 | Basidiomycota | Unassigned | Unassigned | 384/460(83%) | JF908384 | *Mycena acicula* | -- |
| 513 | 4 | 10 | Ascomycota | Helotiales | Unassigned | 580/601(97%) | FJ475721 | Uncultured Ascomycota | Forest soil in Sweden |
|  |  |  |  |  |  | 537/564(95%) | FJ196296 | Helotiales sp. | -- |
| 514 | 2 | 7 | Ascomycota | Dothideales | Saccotheciaceae | 448/488(92%) | KF274441 | Uncultured fungus | -- |
|  |  |  |  |  |  | 463/516(90%) | KJ690089 | *Aureobasidium* sp. | -- |
| 515 | 2 | 23 | Ascomycota | Helotiales | Unassigned | 488/489(99%) | KC965555 | Uncultured fungus | Arctic soil in Canada (76.23N, 119.30W) |
|  |  |  |  |  |  | 301/318(95%) | EF029227 | *Helicodendron triglitziense* ^#^ | -- |
| 516 | 1 | 2 | Basidiomycota | Sporidiobolales | Unassigned | 383/390(98%) | AB178481 | *Sporobolomyces gracilis* ^#^ | Unreported |
| 517 | 2 | 3 | Ascomycota | Helotiales | Unassigned | 378/401(94%) | JX382410 | Uncultured fungus | -- |
|  |  |  |  |  |  | 375/402(93%) | KR063523 | Uncultured Helotiales | -- |
| 518 | 3 | 83 | Unassigned | Unassigned | Unassigned | 448/449(99%) | KC457123 | Uncultured endophytic fungus |  |
| 519 | 9 | 117 | Ascomycota | Unassigned | Unassigned | 391/417(94%) | JF300533 | Uncultured fungus | -- |
|  |  |  |  |  |  | 373/436(86%) | DQ351724 | *Troposporella fumosa* | -- |
| 520 | 1 | 15 | Chytridiomycota | Chytridiales | Chytriomycetaceae | 299/362(83%) | GU065483 | Uncultured fungus | -- |
|  |  |  |  |  |  | 157/164(96%) | AY997049 | *Entophlyctis* sp. ^#^ | -- |
| 521 | 1 | 5 | Basidiomycota | Sporidiobolales | Unassigned | 402/465(86%) | FJ552856 | Uncultured Sporidiobolales | -- |
| 522 | 3 | 44 | Chytridiomycota | Chytridiales | Chytriomycetaceae | 426/510(84%) | JX384003 | Uncultured fungus | -- |
|  |  |  |  |  |  | 169/180(94%) | EU352770 | *Chytriomyces poculatus* | -- |
| 523 | 1 | 1086 | Basidiomycota | Cantharellales | Clavulinaceae | 500/525(95%) | EU819415 | *Clavulina cristata* ^#^ | -- |
| 524 | 2 | 6 | Ascomycota | Hypocreales | Ophiocordycipitaceae | 401/407(99%) | KC237381 | *Elaphocordyceps* sp. ^#^ | Lichen in China (Mt. Qingliang, Zhejiang) |
| 525 | 3 | 133 | Basidiomycota | Thelephorales | Thelephoraceae | 480/481(99%) | EU668945 | Uncultured *Tomentella* ^#^ | Plant roots in forest soil in Estonia |
|  |  |  |  |  |  | 465/467(99%) | JX630533 | *Tomentella lilacinogrisea** | Plant root in Canada (76.21N, 119.28W) |
| 526 | 8 | 98 | Ascomycota | Hypocreales | Nectriaceae | 546/546(100%) | KM889543 | Uncultured *Fusarium* | Farm soil in USA: Illinois |
|  |  |  |  |  |  | 545/546(99%) | KP942940 | *Fusarium oxysporum** | Plant (*Ensete ventricosum*) |
| 527 | 1 | 9 | Rozellomycota | Unassigned | Unassigned | 321/340(94%) | KF296841 | Uncultured fungus (Rozellomycota) | -- |
| 528 | 2 | 3 | Ascomycota | Baeomycetales | Trapeliaceae | 411/417(99%) | KC966233 | Uncultured fungus | Arctic soil in Canada (78.78N, 103.55W) |
|  |  |  |  |  |  | 346/423(82%) | NR119924 | *Placopsis perrugosa* | -- |
| 529 | 6 | 46 | Ascomycota | Thelebolales | Thelebolaceae | 382/389(98%) | JX489808 | Uncultured soil fungus | Soil in China: Helongjiang |
|  |  |  |  |  |  | 382/389(98%) | KC922131 | Uncultured *Thelebolus* ^#^ | Stool in China |
| 530 | 2 | 4 | Ascomycota | Hypocreales | Ophiocordycipitaceae | 400/402(99%) | KM652166 | *Hirsutella* sp. ^#^ | Poland |
| 531 | 2 | 346 | Chytridiomycota | Olpidiales | Olpidiaceae | 547/558(98%) | KF493951 | Uncultured *Olpidium* ^#^ | Farm soil |
| 532 | 2 | 2 | Ascomycota | Helotiales | Leotiaceae | 476/477(99%) | KF296986 | Uncultured fungus | Arctic soil in Canada (76.23N, 119.30W) |
|  |  |  |  |  |  | 440/458(96%) | KC834040 | *Alatospora constricta* ^#^ | -- |
| 533 | 2 | 20 | Basidiomycota | Sebacinales | Sebacinaceae | 415/444(93%) | KC019912 | Uncultured Sebacinales | -- |
| 534 | 2 | 7 | Ascomycota | Helotiales | Dermateaceae | 459/462(99%) | HQ533816 | *Helgardia* sp. ^#^ | Antarctic lake |
| 535 | 2 | 13 | Ascomycota | Unassigned | Unassigned | 334/405(82%) | HQ631052 | Pleosporales sp. | -- |
| 536 | 2 | 26 | Basidiomycota | Atheliales | Atheliaceae | 451/482(94%) | EU490078 | Uncultured Basidiomycota | -- |
|  |  |  |  |  |  | 367/404(91%) | EU118605 | *Athelia pyriformis* | -- |
| 537 | 4 | 12 | Ascomycota | Unassigned | Unassigned | 251/263(95%) | KF296938 | Uncultured fungus | Arctic soil in Canada (76.23N;119.30W) |
|  |  |  |  |  |  | 251/306(82%) | AF096212 | *Umbilicaria rigida* | -- |
| 538 | 3 | 2000 | Ascomycota | Lecideales | Lecideaceae | 431/466(92%) | AM292669 | *Bilimbia microcarpa* | -- |
| 539 | 7 | 73 | Basidiomycota | Agaricales | Cortinariaceae | 599/617(97%) | AY083178 | *Cortinarius atrocoeruleus* ^#^ | unreported |
| 540 | 1 | 77 | Ascomycota | Verrucariales | Verrucariaceae | 369/389(95%) | KC965500 | Uncultured fungus | Arctic soil in USA (69.67N, 148.72W) |
|  |  |  |  |  |  | 267/287(93%) | FJ664867 | *Verrucaria latebrosa* | -- |
| 541 | 1 | 9 | Chytridiomycota | Chytridiales | Unassigned | 287/350(82%) | JX384003 | Uncultured fungus | -- |
|  |  |  |  |  |  | 172/192(90%) | EU352774 | Chytridiales sp. | -- |

*.For sequence similarity ≥ 99%, the species were accepted. #.For sequence similarity between 95% and 99%, only the genus was accepted. §.For sequence similarity ≥ 97%, the originally reported habitats of fungal taxa were accepted.
