# Supplementary material for: Soil pH is a Key Determinant of Soil Fungal Community Composition in the Ny-Ålesund Region, Svalbard (High Arctic)
Source: Front Microbiol. 2016 Feb 26;7:227. doi: 10.3389/fmicb.2016.00227 (PMC4767930; doi:10.3389/fmicb.2016.00227)
Supplement: Supplementary file 3 [file Table_3.DOCX]

**Table S3 | Lefse analysis showing the fungal taxonomic groups which are significantly different among three soil pH levels.**

| Taxonomic rank | Name | pH8-9^#^ | pH7-8^#^ | pH6-7^#^ |
| --- | --- | --- | --- | --- |
| Order | Coniochaetales | - | - | 4.33 (α=0.039) |
| Order | Verrucariales | 5.02 (α=0.010) | - | - |
| Order | Umbilicariales | 4.30 (α=0.044) | - | - |
| Order | Sordariales | - | 4.69 (α=0.018) | - |
| Family | Verrucariaceae | 4.92 (α=0.014) | - | - |
| Family | Tilachlidiaceae | 4.32 (α=0.006) | - | - |
| Family | Nectriaceae | - | 4.21 (α=0.018) | - |
| Family | Hyaloscyphaceae | - | - | 4.37 (α=0.036) |
| Family | Coniochaetaceae | - | - | 4.39 (α=0.039) |
| Family | Herpotrichiellaceae | 4.66 (α=0.021) | - | - |
| Family | Pyronemataceae | - | 4.04 (α=0.042) | - |
| Genus | *Neonectria* |  | 4.53 (α=0.126) |  |
| Genus | *Rhinocladiella* | 4.72 (α=0.003) |  |  |
| Genus | *Nectria* |  | 4.03 (α=0.031) |  |
| Genus | *Alta* | 4.69 (α=0.005) |  |  |

# LDA score with α value for the factorial Kruskal-Wallis test among soil types.
